# Disturbed flow induces a sustained, stochastic NF-κB activation which may support intracranial aneurysm growth *in vivo*

**DOI:** 10.1038/s41598-019-40959-y

**Published:** 2019-03-18

**Authors:** Daniel C. Baeriswyl, Ioanna Prionisti, Tom Peach, Grigoris Tsolkas, Kok Yean Chooi, John Vardakis, Sandrine Morel, Mannekomba R. Diagbouga, Philippe Bijlenga, Simon Cuhlmann, Paul Evans, Brenda R. Kwak, Yiannis Ventikos, Rob Krams

**Affiliations:** 1 Department Mechanical Engineering, University College London, London, UK; 2 Department Bioengineering, Imperial College London, London, UK; 3 SEMS, Queen Mary University London, London, UK; 4 Department Pathology and Immunology, University of Geneva, Geneva, Switzerland; 5 Department Medicine, University of Sheffield, Sheffield, UK; 6 Department Clinical Neurosciences, Geneva University Hospitals, Geneva, Switzerland

## Abstract

Intracranial aneurysms are associated with disturbed velocity patterns, and chronic inflammation, but the relevance for these findings are currently unknown. Here, we show that (disturbed) shear stress induced by vortices is a *sufficient* condition to activate the endothelial NF-kB pathway, possibly through a mechanism of mechanosensor de-activation. We provide evidence for this statement through *in-vitro* live cell imaging of NF-kB in HUVECs exposed to different flow conditions, stochastic modelling of flow induced NF-kB activation and induction of disturbed flow in mouse carotid arteries. Finally, CFD and immunofluorescence on human intracranial aneurysms showed a correlation similar to the mouse vessels, suggesting that disturbed shear stress may lead to sustained NF-kB activation thereby offering an explanation for the close association between disturbed flow and intracranial aneurysms.

## Introduction

The progression and high mortality of chronic arterial diseases - like atherosclerosis and aneurysms - are intimately associated with the activity of pro-inflammatory pathways^1,2^, of which the NF-κB pathway has been extensively studied^3–5^. An intriguing observation is that both atherosclerosis and intracranial aneurysms are spatially confined^6,7^ and ample evidence indicates that patterns of blood flow contribute to this localisation, which raises the question of how the NF-κB pathway is influenced by blood flow.

Shear stress is the drag force (per unit area) imposed on the endothelium by the blood flow which regulates all components of the NF-κB pathway^8–11^, but the nature of the velocity pattern that regulates the NF-κB pathway is still under debate^10,12–15^. According to older studies, stationary low shear stress [0.1–0.5 N/m or Pa] is sufficient to activate the pathway^12,13,15^, whereas other studies indicate that pulsatile shear stress^10,11,15^ and/or disturbed shear stress^11,14^ are essential for its activation.

The reason for this discrepancy is currently unknown, but single cell studies indicate large cell-to-cell variation in NF-κB activity after stimulation with TNF-α^16–19^, which can be described by a model incorporating stochastic receptor activation. We will propose herein that this model of single cell stochastic receptor activation may be an explanation of the above mentioned discrepancy.

While it is currently under debate how shear stress induces NF-κB activity, cultured endothelial cells that are oriented randomly at the start of flow studies display NF-κB activity after application of flow, which gradually disappears when endothelial cells become aligned, a process which takes 12–16 hours^20–22^, and NF-κB activity re-appears when the flow direction is changed^20^. In the intact circulation, endothelial cells are always aligned, except in disturbed flow areas where endothelial cells become randomly dis-aligned to the flow direction^20,21^, and NF-κB activity becomes again upregulated^11,23,24^. The close relationship between endothelial cell misalignment and NF-κB has been reported repeatedly in these studies^11,23,24^.

We hypothesize that the dis-alignment of endothelial cells *in vivo* and *in vitro*, leads to stochastic mechanosensor activation and thereby to NF-κB activation. To support this concept, we show that the single cell, stochastic behaviour of the NF-κB pathway to flow is initiated early after flow initiation, i.e where endothelial cells are known to be randomly oriented, or in disturbed flow regions in the intact circulation, thereby reconciling above mentioned literature. Furthermore, we show that, in intact murine vessels, the induction of disturbed-flow-induced NF-κB activation leads to monocyte recruitment and a sterile inflammation^11^. We also demonstrate that, in human intracranial aneurysm, disturbed flow associates with randomly aligned cells and high NF-κB activation. Finally, we confirm that this activation of the NF-κB pathway in randomly dis-aligned cells is well described by a mathematical model of the NF-κB pathway incorporating stochastic receptor de-activation.

## Single Cell Measurements of Nuclear Translocation of NF-κB in HUVECs

**Figure 1:**
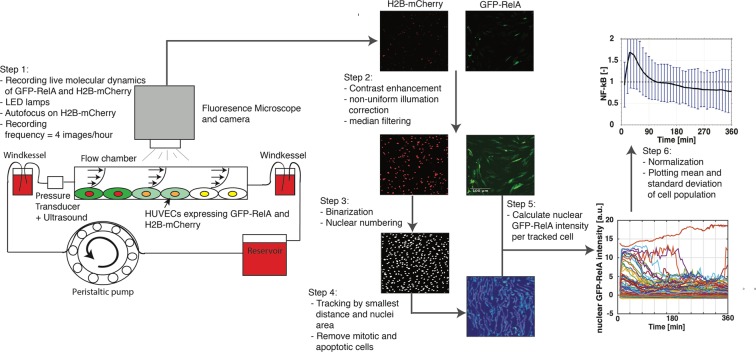
Schematic diagram of the experimental pipeline: Step 1: Perfusion system including flow chamber, two windkessels, medium reservoir and a peristaltic pump. Cells within the flow chamber were recorded with the fluorescence microscope using LED lamps. Step 2: The raw image of H2B-mCherry and GFP-RelA were processed by enhancing the contrast, correcting non-uniform illumination and removing noise with a median filter. Step 3: H2B-mCherry image was made binary and the nuclei were numbered. Step 4: The numbered nuclei were tracked throughout all time frames. Step 5: The coordinates from the tracked nuclei were used to calculate the nuclear GFP-RelA intensity in the corrected GFP-RelA image. Step 6: nuclear GFP-RelA intensity in each cell was normalized by the time average GFP-RelA intensity. The population mean of the normalized nuclear GFP-RelA intensity including standard deviation was plotted as the result.

Temporal dynamics of NF-κB in individual cells were measured in HUVECs transfected with GFP-RelA and H2B-mCherry (Sup. Figs 1 and 2) treated with TNF-α, untreated or exposed to shear stress. Note that we will use NF-κB and RelA interchangeably throughout the paper. As endothelial cells are difficult to transfect, a bespoke electroporation protocol was developed yielding >70% transfection rates (see methods and Sup. Fig. 3), enabling us to record temporal dynamics in large numbers of endothelial cells in each experimental condition using high-throughput wide-field fluorescence microscopy imaging. We designed an experimental pipeline that enables live recording of spatial location of NF-κB in single endothelial cells within a large population exposed to flow conditions. The experimental pipeline including tissue culture protocols, imaging, image pre-processing, tracking algorithms and quantification is depicted in Fig. 1, described in the methods section and more in depth and complementary material is listed in the Supplementary Material (Sup. p.1–13).

## Endothelial Cells Display Similar NF-κB Dynamics as Other Cells to TNF-α

**Figure 2:**
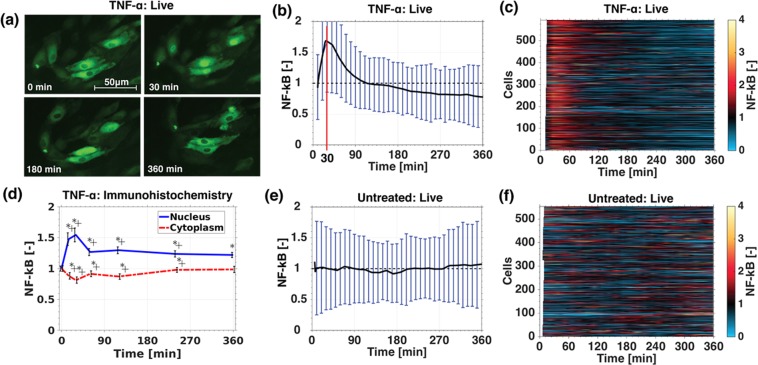
TNF-α stimulated nuclear translocation of NF-κB in HUVECs. HUVECs transfected with GFP-RelA and H2B-mCherry were stimulated with 10 ng/mL TNF-α **(a**–**c**). A time series of GFP-RelA shows strong nuclear concentration at 30 minutes and empty nuclei at 0 and 360 minutes **(a)**, the population mean of approximate 600 single cell measurements **(b)**, projected view of the entire cell population **(c)**. Immunohistochemistry of non-transfected HUVECs treated with 10 ng/mL TNF-α fixed and stained (p65-AF488) at different time points point normalized to unstimulated cells (*p < 0.05 = significant change to unstimulated cells, ^**+**^p < 0.05 = significant change to previous time point) **(d)**. The population mean of approximately 600 single untreated cells **(e)** and projected view of the entire population **(f)** of untreated GFP-RelA and H2B-mCherry transfected HUVECs recorded for 360 minutes.

With the experimental pipeline dsescribed above we measured the nuclear translocation of GFP-RelA in a large population of cells exposed to 10 ng/mL TNF-α (Fig. 2a–c, Sup. Figs 17 and 18). The population of endothelial cells responded homogenously to the high TNF-α concentration, with a nuclear GFP-RelA intensity of all cells peaking at approximate 30 minutes (Fig. 2a–c) similar to that shown in other cell types^16–19^. Clear and bright nuclei can be observed at 30 minutes while at 0 and 360 minutes the nuclei appear almost empty (Fig. 2a). A projection map of all individual cells and a population average confirms the single cell experiments, by showing the strong nuclear translocation in response to TNF-α after 30 minutes of treatment (Fig. 2b,c). The single cell signals were normalized by their time average intensity (Sup. p.12). Time average normalization was found to be less noise sensitive (e.g. in comparison with normalization by the first-time point), while not affecting temporal deviations from the mean. Increasing the TNF-α concentration to 100 ng/mL increased single cell synchronization (Sup. Figs 24e and 25e) and decreasing to 1 ng/mL TNF-α (Sup. Figs 24g and 25e) resulted into a weak amplitude of nuclear translocation at 30 minutes, confirming previous studies^19^. To further confirm the single cell dynamics, immunohistochemistry (IHC) of non-transfected HUVECs exposed to 10 ng/mL TNF-α was performed at various time points (Fig. 2d). Non-transfected HUVECS were stained for RelA (p65). The TNF-α treated nuclear intensities were normalized by the nuclear intensity of non-treated cells (Sup. p.7). Initially treated and non-treated signals were equal, resulting in a normalized NF-κB value of one. IHC confirmed a peak of nuclear RelA concentration at around 30 minutes with a 1.5 stronger intensity signal in comparison to the control. This validates the single cell measurements. IHC confirmed a peak of nuclear p65 (RelA) concentration at around 30 minutes, validating the single cell measurements.

Unstimulated transfected HUVECs appeared to be very heterogeneous with a minority of cells experiencing randomly occurring nuclear translocation of GFP-RelA (Fig. 2f). This agrees with a previous study that observed significant random nuclear translocation in 15% of the cell population^16^. The nuclear GFP-RelA population mean of unstimulated HUVECs resembled its initial, zero value (Fig. 2e).

## Single Cell Meassurements Unveiled a Flow Dependent NF-κB Activity Level

**Figure 3:**
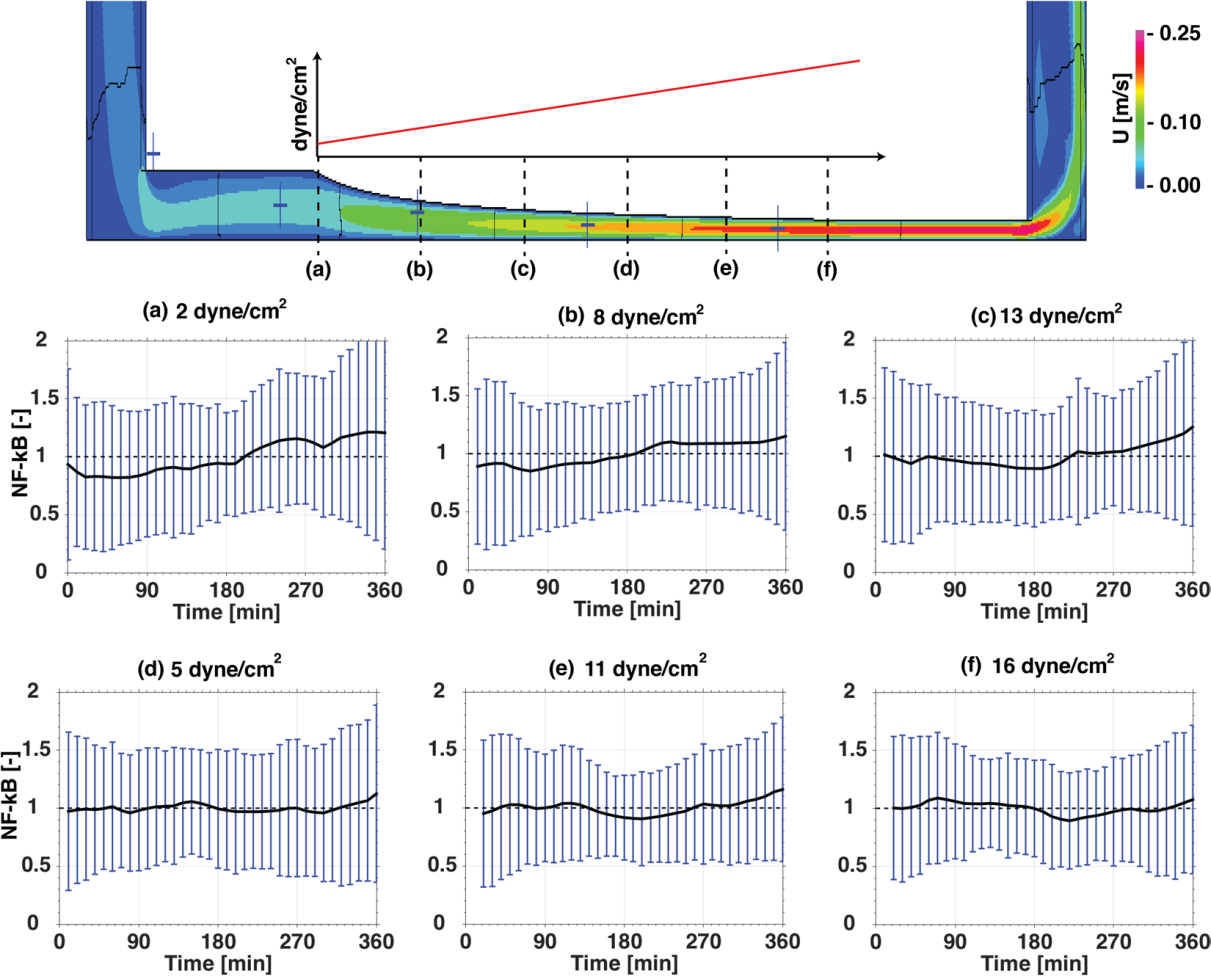
Effect of a spatial gradient on NF-κB: The velocity profile of the gradient channel obtained from CFD simulations. The ramp like structure creates a linear increasing shear stress (depicted with an arbitrary plot) at the bottom wall of the channel. The nuclear GFP-RelA intensity at six different shear stress magnitude positions (**a** - 2 dyne/cm^2^, **b** - 5 dyne/cm^2^, **c** - 8 dyne/cm^2^, **d** - 11 dyne/cm^2^, **e** - 13 dyne/cm^2^, **f** - 16 dyne/cm^2^) indicated on the velocity profile were recorded with an average cell count of 300 per position in three repeats.

**Figure 4:**
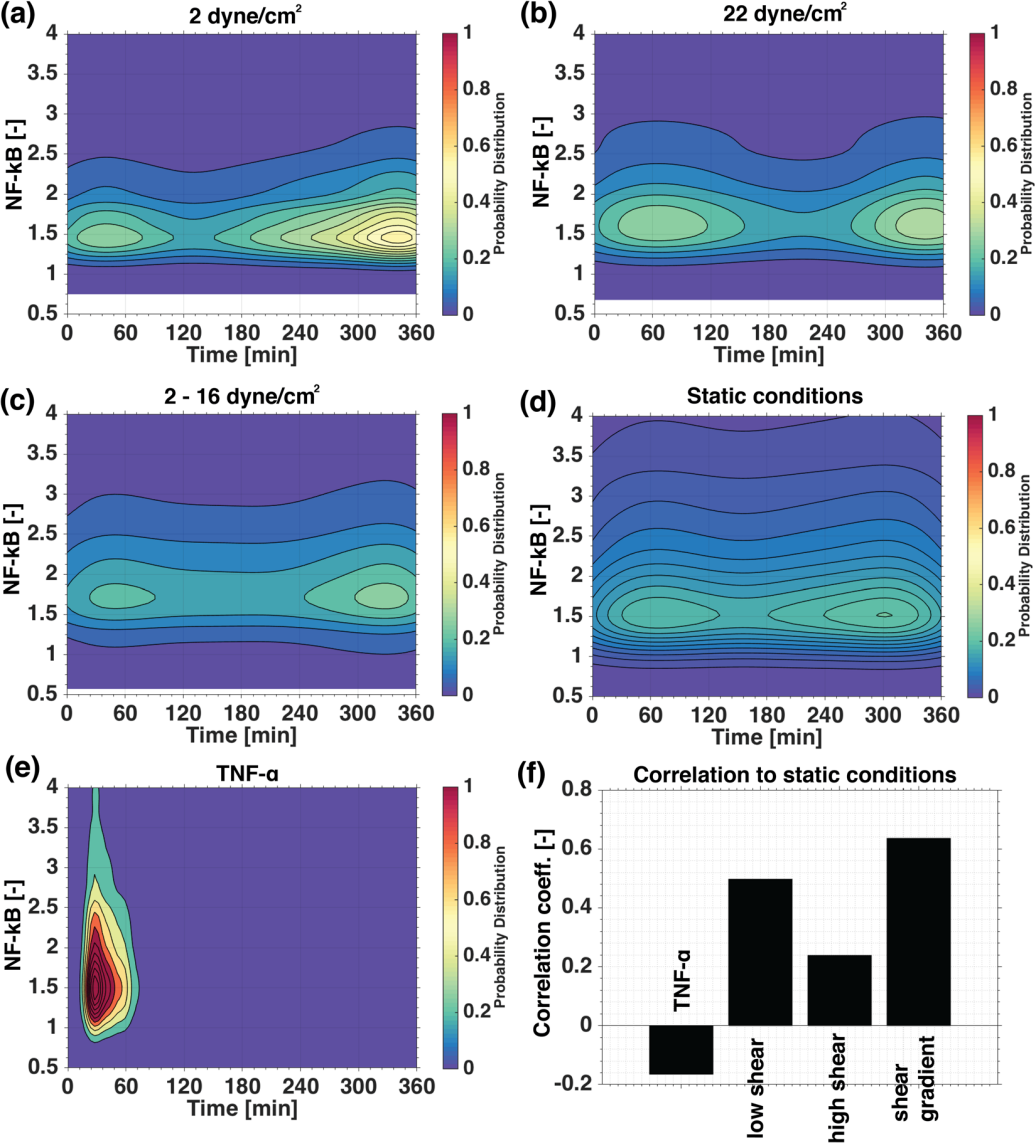
The effect of shear stress on the distribution of nuclear NF-κB peaks. The maximum peak probability distribution of NF-κB time series measured in HUVECs exposed to: uniform low shear stress (2 dyne/cm^2^) (1312 cells) (**a**), uniform high shear stress (22 dyne/cm^2^) (1789 cells) (**b**), shear stress gradient (2–16 dyne/cm^2^, measured at 2, 5, 8, 11, 13, and 16 dyne/cm^2^) (1676 cells) (**c**), static conditions (554 cells) (**d**), and after 6 hours of 10 ng/mL TNF-α (594 cells) (**e**). Plots were generated by calculating the normal distribution in time and intensity of all single cell peaks of the normalised NF-kB value for each experimental condition. Peak refers to the highest value of the normalised NF-kB time serie of a single cell. The colour indicates the NF-kB peak distribution density of the population in time and intensity. Finally, we display the ratio of peak probability distribution of each intervention to static conditions. TNF-α resulted in a low negative correlation, while under flow, high shear stress affected the distribution of peaks the most (**f**).

We exposed HUVECs to different levels of constant shear stress, and to a spatial gradient of shear stress. The latter was imposed through a newly developed flow chamber (Sup. p.13–16). The average, temporal response of NF-κB in endothelial cells was minimally affected by the shear stress perturbation, albeit with a notable, large variability around the mean (Fig. 3). To further elucidate the underlying mechanism of this large variability, we examined single cell NF-κB peak intensity distributions in large numbers of cells (500 to 1200 cells, Fig. 4).

We firstly confirmed that this approach leads to a strong synchronisation of individual cells with an early aggregate of peak intensity NF-κB values in response to a high dosage of 10 ng/mL TNF-α (Fig. 4e). A more heterogeneous response was observed under static, non-shear stress conditions, giving a broad dispersion of aggregates (Fig. 4d). However, two low intensity peaks (5x smaller than with TNF-α stimulation) under static conditions at approx. 60 and 300 minutes were observed, suggesting a low level of synchronicity at these points in time^16^. Subsequently, we imposed shear stress and demonstrated that, at low levels of shear, shear stress-induced early and late aggregates of peak intensity values of NF-κB varied with shear stress levels. High shear stress induced stronger early peak intensity aggregates (Fig. 4a–c). This analysis indicates that patterns in NF-κB activity were present at a single cell level in HUVECs exposed to shear stress, which were not identified by the average response analysis.

## Clustering Identifies Groups of Cells With Different Temporal NF-κB Activity

To better quantify patterns in single cell NF-κB dynamics, *Kmeans* clustering was chosen as the best method to proceed for three reasons: firstly, it confirmed the random pattern in unstimulated cells (Sup. Fig. 21c,d). Secondly, it confirmed a uniform response with variable intensity after TNF-α stimulation (Sup. Fig. 24a–d). Lastly, it created reproducible results after constant, low and high shear stress values (Sup. Fig. 21e–h), while other clustering methods (hierarchical clustering, thresholding or pulse detection) produced a variable response.

**Figure 5:**
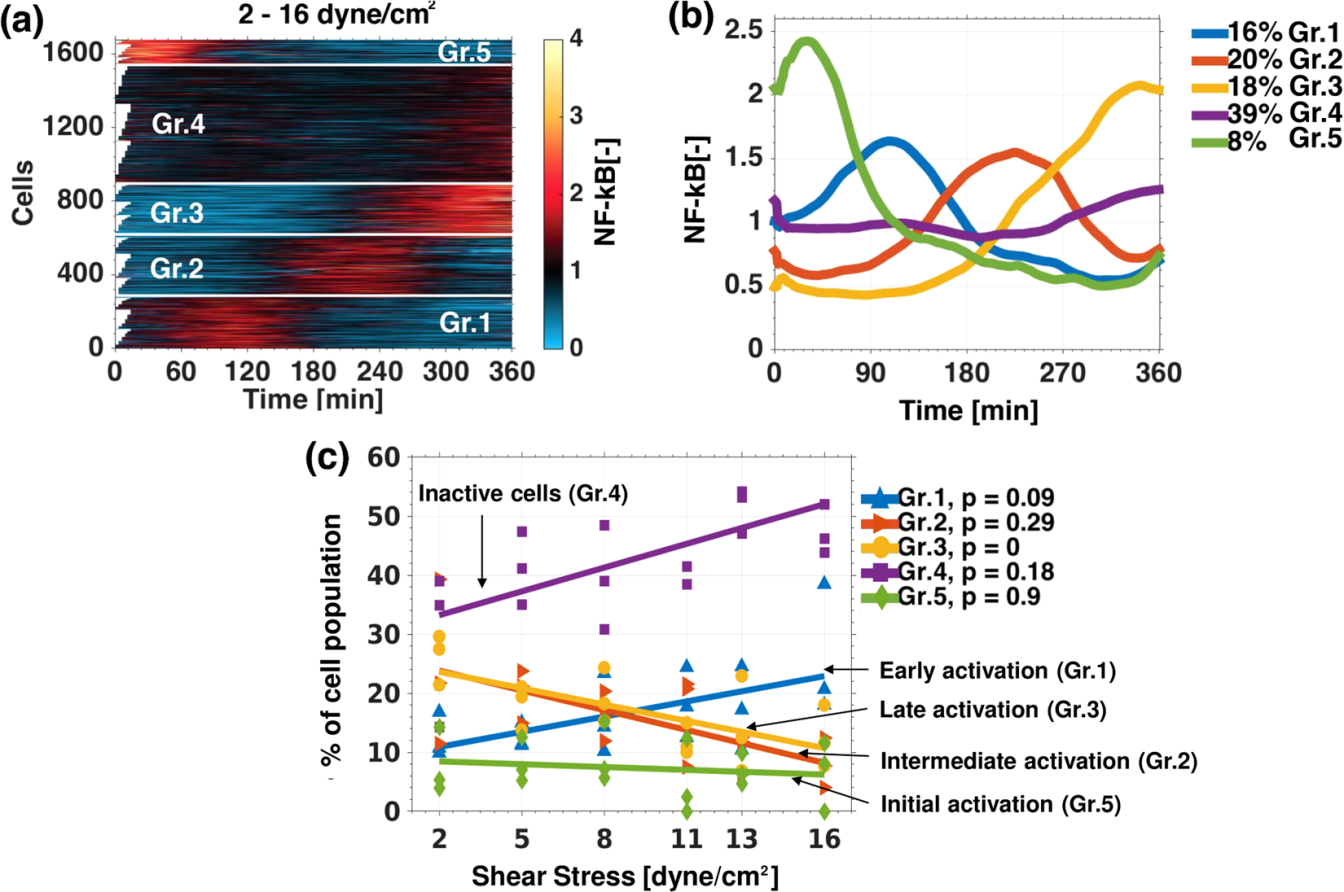
Kmeans clustering identifies groups of endothelial cells with specific NF-κB dynamics. Kmeans cluster map of the nuclear GFP-RelA intensity time series normalized by time average of HUVECs exposed to a shear stress gradient of 2–16 dyne/cm^2^. **(a)** Means of the cluster groups (Gr.1 = blue, Gr.2 = red, Gr.3 = yellow, Gr.4 = violet, Gr.5 = green) are displayed in time. **(b)** The change in population percentages of each cluster group at different shear stress positions within the gradient channel fitted to a linear regression. P-value is calculated with an ANOVA. Same colors as in plot b **(c)**.

Clustering of single cell time series obtained from the shear stress gradient channel (Fig. 5a) identified five groups of cells: Early activation (Gr.1, blue), intermediate activation (Gr.2, red), late activation (Gr.3, yellow), inactive cells (Gr.4, purple) and initial activation (Gr.5, green) (Fig. 5a–c). Approximately 60% of the cell population was characterised as having NF-κB pathway activation in the shear stress gradient. The population percentage of all groups (active and inactive), except the immediate activation group, changed with the level of shear stress (Fig. 5c). The increments in shear stress increased the early responsiveness of NF-κB to flow over slow responses. At high shear stress magnitudes, early activation (Gr.1) counted approximately 25% of the cell population, while most cells were inactive counting approximately 50% of the population. At low shear stress magnitudes, the intermediate and late activation groups (Gr.2 and 3) were predominantly present with approximately 25% each and only 30% were considered inactive (Fig. 5c). Hence, different NF-κB single cell behaviours were observed under shear stress, and the local magnitude of shear force increased or decreased the percentage of such.

## Stochastic Modelling of Receptor Activation Reproduces Cellular Heterogeneity and Predicts Increased NF-κB Activity in Disturbed Flow

To describe the underlying noisy mechanosensor based de-activation mechanism of the single cell NF-κB pathway, we extended a previous NF-κB model^25^. Detailed description of the model is in the Supplementary Material (Sup. p20–40).

First, a simplified stochastic TNF-α receptor model was created to reproduce the TNF- α response as observed in Fig. 2. This model could describe a population response of single cells to different TNF-α doses with high agreement to experimental measurements (Sup. Figs 24 and 25). Cell heterogeneity could be reproduced and random activation of NF-κB under static conditions led to a large standard deviation (Sup. Fig. 25h) as observed in measurements and in other studies^16^. Noisy dynamics were demonstrated for other members of the NF-κB, i.e. IKK and IκBα in static conditions and at low TNF-α dosages (Sup. Figs 26 and 27).

**Figure 6:**
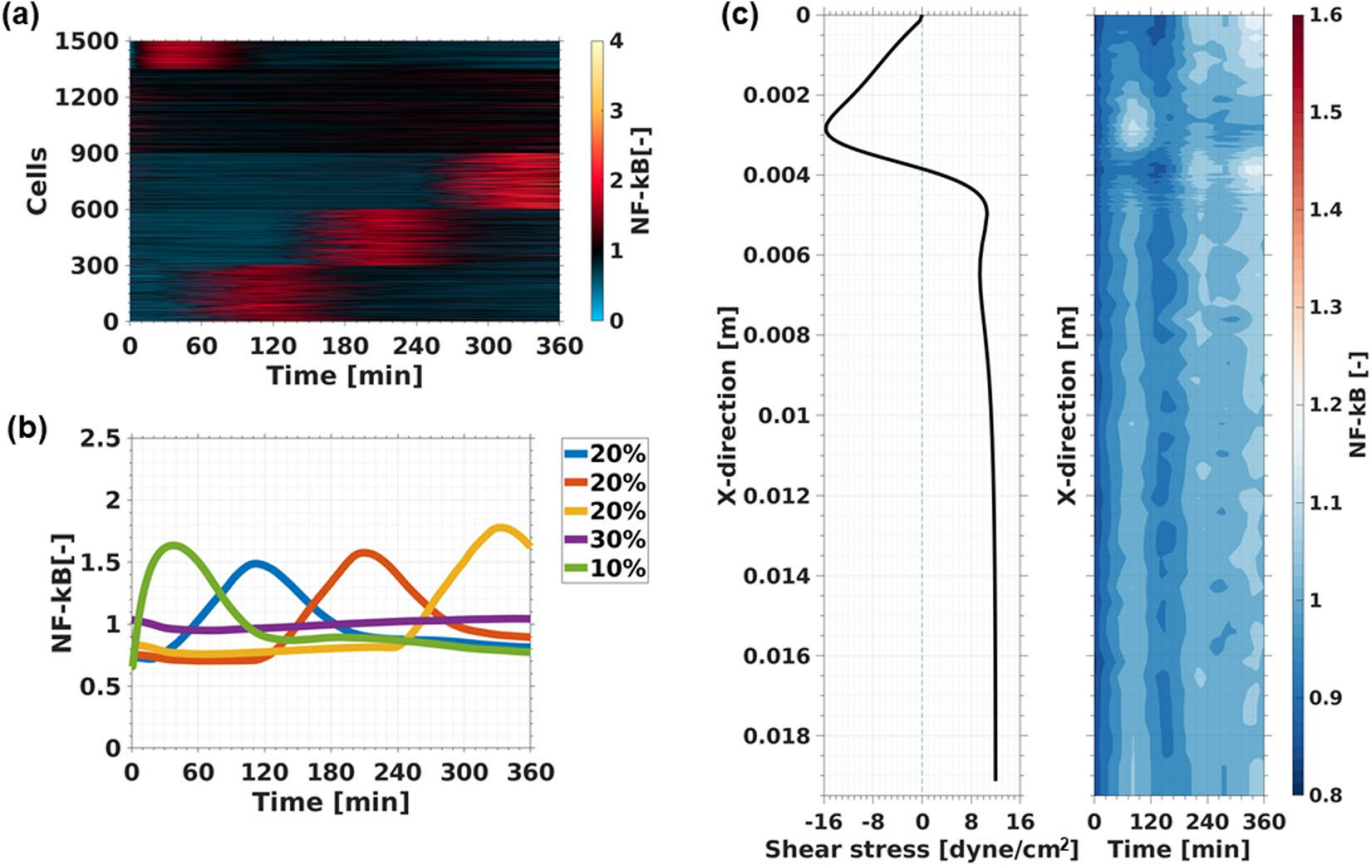
Flow induces NF-κB activation: Cluster map of the predicted temporal nuclear NF-κB concentration (**a**) of a cell population exposed to a shear stress gradient of 2–16 dyne/cm^2^. Means of the predicted temporal nuclear NF-κB concentration (**b**) of each cluster group. The shear stress profile of the bottom wall after a sudden expansion (detailed CFD model description in Sup. Material) is depicted (**c**) and next to it, the temporal nuclear NF-κB changes in a cell population of 50,000 cells **(d)** exposed to the stationary shear stress profile from panel c.

Next, the model was extended with a stochastic mechanosensory receptor mechanism (Sup. p32–40). The formulae and description of the model can be found in the Supplementary Material. In Fig. 6, this model clearly demonstrates great similarities of nuclear NF-κB dynamics between the model (Fig. 6a,b) and the experimental conditions (Fig. 5a,b) for cells exposed to a shear stress gradient of 2–16 dyne/cm^2^. The model predicts each group and their specific time dynamics. This suggests that receptor de-activation may play a role in flow dependent NF-κB activation. More detailed shear induced NF-ΚkB pathway dynamics predictions are depicted in the Supplementary Material (Sup. Figs 30–36).

In Fig. 6c,d the stochastic NF-κB model was applied to a 2D-backward facing step channel geometry which creates a disturbed flow pattern. Detailed CFD simulations to obtain the shear stress profile are shown in the Supplementary Material (Sup. p.42). The model predicts that disturbed flow (vortex formation) is a sufficient condition for early NF-κB activation. The disturbed flow region, where shear stress of −16 dyne/cm^2^ occurs, shows the highest NF-κB intensity within the channel at 30 minutes. Previous experimental studies reported elevated nuclear NF-κB concentration at disturbed flow areas in a step channel after 30 minutes^14^ and 24 hours^26^. However, our model predicts that persistant and constant vortex formation (longer than 300 minutes) leads to strong NF-κB activation outside the high shear stress regions. This might indicate that short living disturbed flow regions initiate immediate NF-κB activation, and stagnant or low shear stress regions develop a slow and sustained NF-κB activation. Prediction of the effect of disturbed flow on major proteins (IKK and IκBα) of the NF-κB pathway is presented in the Supplementary Material (Sup. Fig. 36).

## Induction of Disturbed Flow in Murine Vessels is Sufficient for NF-κB Activation and Macrophage Uptake

**Figure 7:**
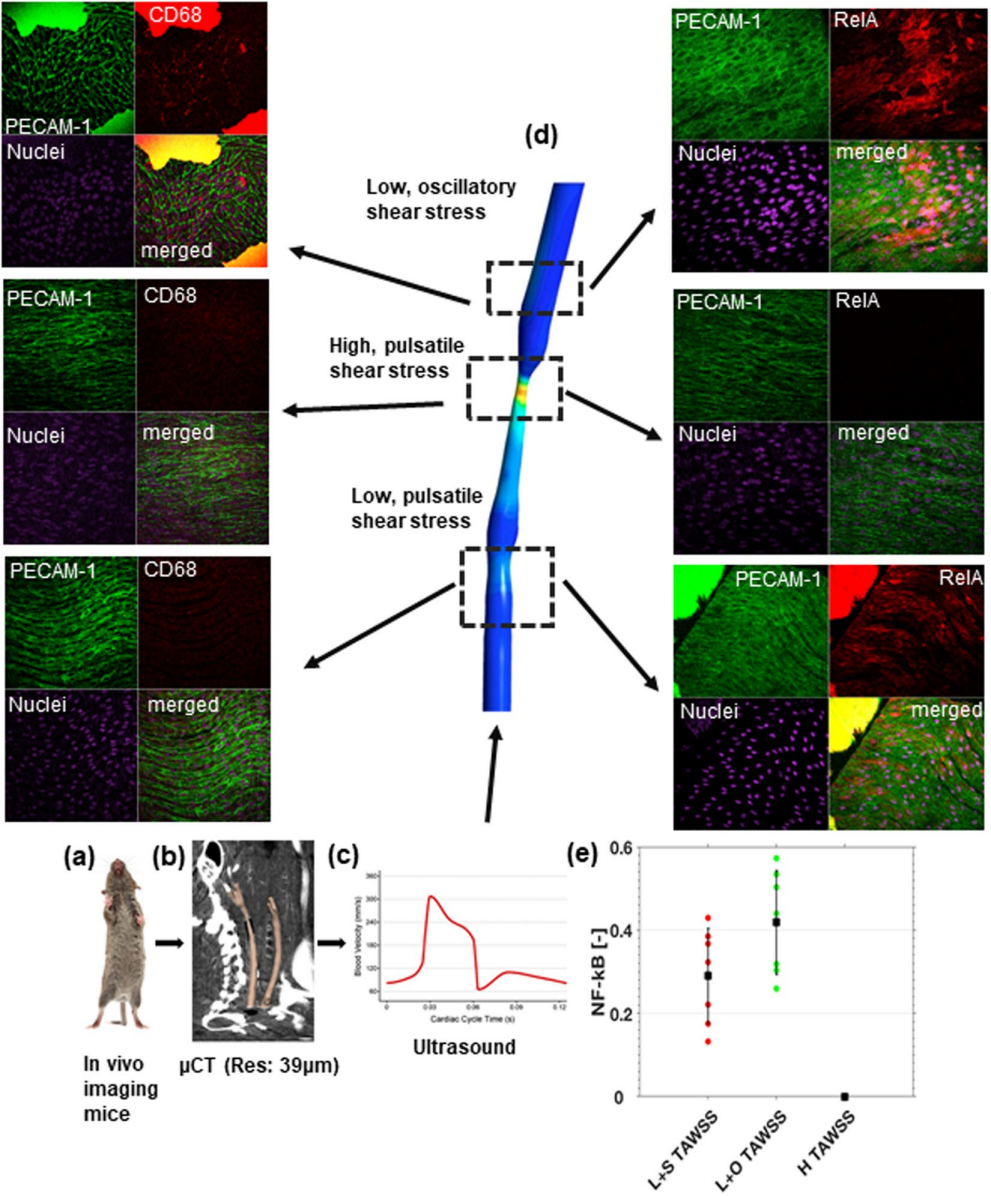
*In vivo* experiments showing that sustained vortex formation is sufficient for NF-κB activation. The carotid arteries of a mouse are imaged with an ultrahigh resolution uCT (**a,b**), and segmented (**b**). The doppler signal of the mouse carotid artery is recorded (**c**) and fed in as boundary conditions for the CFD simulation of the geometry reconstructed from the carotid artery uCT (**b**). Displayed is the time average wall shear stress (TAWSS) from the cuffed vessel (**d**). Panel d shows experiments where CFD is coupled to endothelial cells exposed to low pulsatile shear stress, high pulsatile shear stress, and low oscillatory shear stress. The stains on the right shows PECAM1 for endothelial cell boundaries in the upper left corner, RelA for NF-κB in the upper right corner, a nuclear stain in the lower left corner and all 3 merged in the lower right corner. It is clearly shown that RelA is high in the disturbed velocity region, which indicates high NF-κB activity. Stains on the left are similar to those on the right with the exception that RelA has been changed to CD68 to identify macrophages. For the analysis, five animals with >30 region of interest per animal were analyzed (**e**). NF-κB activity in regions of low shear stress and low disturbed shear stress were both significantly increased with respect to high shear stress regions (p < 0.05). All measurements were normalized to the high shear stress regions. Note that endothelial cells are rounded in the disturbed flow region and elongated in the high shear region. The low shear region shows more aligned but shorter endothelial cells. The picture of the mouse was made by SachaBurkhard@shutterstock.

We tested the model predictions by implanting a tapered external cuff around murine carotid arteries to induce vortices for seven days (Sup. p52). This method has been extensively validated by others and us^25,27–32^ and shown to produce high shear stress in the cuff, low and uniform shear stress upstream of the cuff and disturbed flows (vortices, oscillatory shear stress) downstream of the cuff^25,27–32^. We have developed a method to image the vessels with ultra-high resolution (μCT: isotropic resolution of 40μm), 3D segmentation using level-set methods, and CFD (Fig. 7a–d). Seven days after cuff placement, an *en face* inspection of the murine carotid artery showed that endothelial cells became rounded and lose their elongated, directional geometry as in high shear stress regions (Fig. 7d). Staining for p65 indicated that low shear stress and disturbed shear stress, but not high shear stress increased NF-κB activity (p < 0.05; Fig. 7d,e). Importantly, the increased NF-κB activity leads to increased monocyte/macrophage recruitment to the vessel wall and a sterile inflammation in the disturbed region only (Fig. 7d). Hence, these observations support the *in vitro* observation that vortex formation leads to rounded endothelial cells, which stochastically increase NF-κB activation and leads to macrophage uptake *in vivo*.

## Reduced and Disturbed Shear Stress in Human Aneurysms is Associated with Increased NF-κB Activation

**Figure 8:**
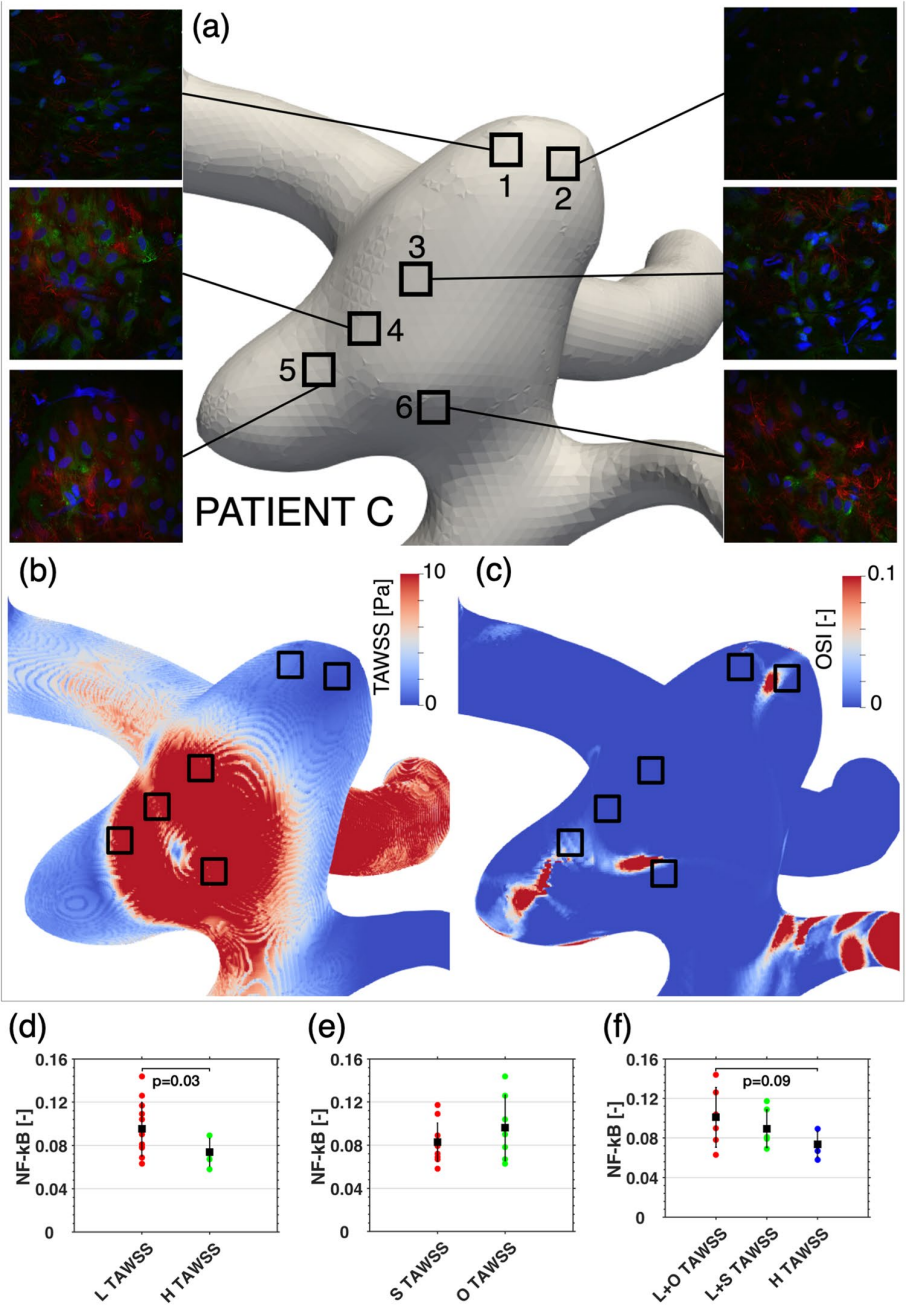
Illustrative example of histology sample locations on the aneurysm of Patient C, stained for RelA (green), PECAM-1 (red) and nucleus (blue) **(a)**, and corresponding in-silico distributions of TAWSS **(b)** and OSI **(c)**. Comparison of sample NF-κB intensities [n = 18] across all three aneurysms with corresponding means and standard deviations indicated in **(d)** for High(H) [n = 6] and Low(L) [n = 12] TAWSS; **(e)** for Stationary(S) [n = 11] and Oscillatory(O) [n = 7] TAWSS; and **(f)** for Low and Stationary(L + S) [n = 6], Low and Oscillatory(L + O) [n = 6], and High(H) [n = 6] TAWSS. Where “High” TAWSS corresponds to TAWSS > 10 Pa and “Oscillatory” TAWSS corresponds to OSI>0.01. Statistical analysis was performed with a t-test (panel d and e) or an ANOVA (panel f) after Shapiro-Wilk tests confirmed no significant departure from a normal distribution (p = 0.05).

Four intracranial aneurysms in separate patients were imaged with 3D rotational angiography, the aneurysms and surrounding vasculature were reconstructed and meshed for CFD analysis in CFD-ACE+ (ESI Group, Paris). Details of the pre-processing pipeline, solver setup and boundary conditions are provided in the methods and Supplementary Material (Sup. 49–52). Pulsatile CFD simulations were performed with average flow rates, and yielded time-averaged wall shear stress (TAWSS), and oscillatory shear index (OSI) distributions that indicate disturbed velocity regions in the aneurysm domes, as shown in Fig. 8a–c.

**Figure 9:**
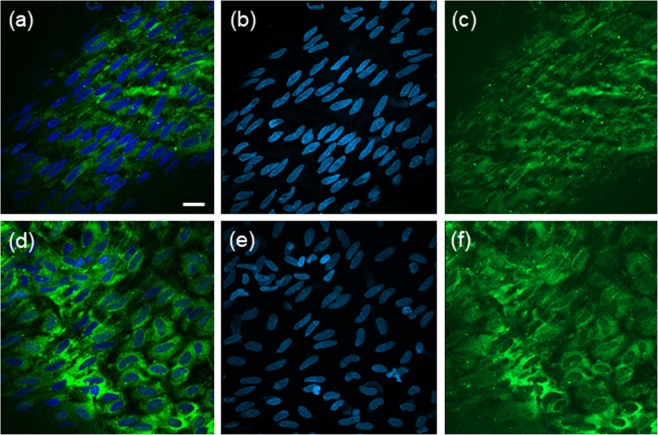
Representative examples of aligned (**a**–**c)** and dis-aligned (**d**–**f**) endothelial cells covering the intraluminal regions of a human intracranial aneurysm. Endothelial cells have been immunolabeled for NF-κB (RelA, in green) and nuclei were counterstained with DAPI (in blue). (**a**,**d**) Are confocal images obtained from one plane. (**b**,**c**,**e**,**f**) Images are maximal projections of combined confocal z-stacks. Scale bar represents 20 mm.

Next, the domes were resected and the tissue was stained for RelA (see methods) and studied in detail with fluorescence confocal microscopy. Images were taken from all regions containing endothelial cells by an observer blinded for the outcomes of the CFD analysis. Regions with aligned (Fig. 9a–c) and dis-aligned endothelial cells (Fig. 9d–f) could be readily recognized in the same sample. Bespoke analysis software analysed all *en face* staining in all images and values were linked to *in silico* TAWSS and OSI, and the results are shown in Fig. 8d–f. Low shear stress clearly identified regions with high NF-κB activity (p < 0.05), as did disturbed, low shear stress (p = 0.09) (Fig. 8d–f).

## Discussion

The major findings are: (i) the high heterogeneity in the response of NF-κB to flow in (cultured) endothelial cells warrants single cell analysis (ii) a mathematical cellular model incorporating mechanosensor de-activation simulates the endothelial cell heterogeneity very well, (iii) this model predicts that flow disturbances (vortex formation) is sufficient for NF-κB activation, (iv) vortex induction in mouse vessels confirmed these predictions and indicated that the NF-κB activation may lead to macrophage accumulation, and (v) disturbed flow in cerebral aneurysm was associated with NF-κB activation. Together, these experiments offer an explanation for the close relationship between flow and aneurysm growth.

Previous flow studies showed increased NF-κB activity after 30 and 60 minutes of high shear stress in different types of endothelial cells using immunohistochemistry^33–35^. Only a few groups studied temporal NF-κB dynamics under flow; in those studies, early and late peak oscillations in NF-κB were measured, in agreement with our data^36^. Our study extends previous studies by showing that these dynamics increase with shear stress and can be interpreted by a mechanism based on variable mechanosensor activation, possibly related to endothelial cell misalignment to the alleged velocity direction (Figs 7 and 9). In this mechanism, endothelial cells in low shear stress environments are either unstimulated, or weakly stimulated leading to slow dynamics, while at high shear stress strongly activated endothelial cells dominate, which show fast dynamics.

The mathematical model, which incorporates NF-κB dynamics and a stochastic receptor density per cell showed strong similarity with the above findings and provided evidence that vortex formation is a sufficient condition for NF-κB activation. These predictions were tested in mouse carotid arteries, where vortices were induced by a cuff. Strong similarity between the model predictions and the measurements were noted, providing evidence that vortex formation is a sufficient condition for NF-κB activation in intact blood vessels. In addition, the NF-κB activation was of such magnitude that it was associated with macrophage accumulation, suggesting that vortices can induce a degree of “sterile inflammation”.

Whether this mechanism is observed in human pathology was evaluated in three intracranial aneurysm domes, which had been exposed to both disturbed and undisturbed velocity profiles. Here, an association between disturbed flow and NF-κB was measured. As these velocity patterns are persistent, we postulate that this may lead to a chronic local inflammation with continuous macrophage recruitment, MMP production and weakening of the vessel wall, leading to continuous growth of the dome and possibly rupture of the intracranial aneurysm.

The new chamber enables to create a linear increase in shear stress by introducing complex gradients. While we previously tested for possible transport processes by perfusing the chamber in two directions^37^, we cannot exclude the possibility that these gradients exert a local effect on the endothelial cells, as suggested by^38^ and our outcomes need to be interpreted with caution.

## Methods

### Cell culture

Human umbilical vein endothelial cells (HUVECs, *PromoCell*, *Heidelberg*, *Germany*) were maintained in endothelial cell growth medium (*PromoCell*, *Heidelberg*, *Germany*). HUVECs were incubated at 37 °C and humidified with 5% CO_2_.

### Plasmid DNA purification

GFP-RelA and H2B-mCherry (*AddGene*, *MA*, *USA*) were received in *E. coli* and stored in agar stab. GFP-RelA was a gift from Warner Greene (Addgene plasmid # 23255) and H2B-mCherry was a gift from Robert Benezra (Addgene plasmid # 20972). The cultures were streaked on a 10 mm culture dish (*Corning*, *MA*, *USA*) containing LB agar medium (*Merck*, *Darmstadt*, *Germany*) using a 10 μL inoculation loop (Sigma Aldrich, MO, USA). Antibiotic selection was performed: Kanamycin (50 μg/mL) for GFP-RelA and Ampicillin (100 μg/mL) for H2B-mCherry. Plates were left for 18 hours at 37 °C in a bacterial incubator (*Labnet*, *NJ*, *USA*). Following initial incubation, a single pure colony was selected and inoculated into a 15 mL snap cap tube (BD Falcon, CA, USA) with 10 mL LB agar medium (*Merck*, *Darmstadt*, *Germany*) supplemented with the appropriate antibiotic and transferred to a rotating incubator (*Weisstechnick*, *Loughborough*, *UK*) at 37 °C for 18 hours. Subsequently, the 10 mL culture was transferred to a flask with 1 L LB agar medium, which contained the appropriate antibiotic for each plasmid and was left to grow for 18 hours in a rotating incubator at 37 °C. The following day the plasmids were isolated and purified from the 1 L culture using the PureLink HiPure Plasmid Megaprep Kit (*ThermoFisher Scientific*, *MA*, *USA*) according to the manufacturer protocols. Subsequently, the plasmids were stored at −20 °C for later usage. The plasmids were sequenced (GATC Biotech, *Constance*, *Germany*), confirming the absence of mutation.

### Transfection

Confluent HUVECs in a tissue culture flask were placed in suspension by incubation in trypsin for 2 minutes at 37 °C. Supernatant was added to neutralise the trypsin and the cell suspension was centrifuged at 280 RCF for 5 minutes at 37 °C. The supernatant was removed, and the cell pellet was used for transfection using a NEON transfection system according to the manufacturer’s protocol. The most efficient and successful transfection setting found was: two 20μs pulses at 1000 V. Detailed protocol in Sup. material (Sup. p.2).

### Flow experiments

A perfusion system was constructed to subject cells grown in flow channels to well-controlled shear stresses (Sup. Figs 4–6). The perfusion system consisted of a media reservoir, two dampers, a peristaltic pump Perista SJ-1220 (*Atto*, *Tokyo*, *Japan*), silicone tubing (*Colepalmer*, *London*, *UK)*, a disposable blood pressure transducer (*AdInstruments*, *Oxford*, *UK*), an ultrasound flow meter (*Transonic*, *NY*, *USA*) and the corresponding flow chambers (Sup. Figs 7 and 8). A custom-made shear stress gradient flow chamber was manufactured, the process is described in detail in the Sup. material (Sup. p.11–14). The media reservoir and dampers were custom designed and externally fabricated (*Cambridge Glass Blowing*, *Cambridge*, *UK*). Drawing for the damper and reservoir can be requested at the corresponding author. The flow rate was measured with an ultrasound flow meter system (*Transonic*, *NY*, *USA*) using a 4PSB probe. The pressure was measured using a disposable blood pressure transducer (*AdInstruments*, *Oxford*, *UK*) and the signal was amplified with a custom-made amplifier. The signal was fed via an Arduino UNO R3 (*Arduino*, *Italy*) into a computer and live monitored in MATLAB (*MathWorks*, *MA*, *USA*). Flow rates and pressure were measured prior to the experiments (Sup. Figs 5 and 6). The uniform low shear stress was set to 2 dyne/cm^2^, uniform high shear stress was 20 dyne/cm^2^ and the shear stress gradient had a range from 2–16 dyne/cm^2^.

### Immunohistochemistry

Following the experiments, cells were immediately washed with PBS, fixed with 4% PFA, permeabilized (0.1% Triton-X in PBS), washed with PBS and then blocked for unspecific binding with 0.5% Bovine Serum Albumin (BSA) for 12 minutes. After blocking, the primary antibodies were added according to the manufacturer’s protocol. The antibodies included: NF-κB p65 (F-6) Alexa Fluor 488, IκBα (H-4) Alexa Fluor 647, p-IKKα/β (Ser 180/Ser 181) (*Santa Cruz Biotechnology Inc*., *CA*, *USA*), and Alexa Fluor 568. Nucleic acids was stained with HOECHST 33342 (*Thermo Fisher Scientific*, *MA*, *USA*).

### Image acquisition immunohistochemistry

Immunohistochemistry images were acquired with a Zeiss Axio Observer wide-field microscope (*Zeiss*, *Oberkochen*, *Germany*) using a 20x magnification lens. Sequential scanning with the appropriate wavelengths according to the fluorophores’ spectrums was performed.

### Image acquisition live cell imaging

Live cell experiments were performed with the wide-field microscope. After placing the sample in the holder at the microscope, the tubing, media reservoir and dampers were placed inside the heating box (37 °C). An external gas supply (air with 5% CO_2_) was connected directly to the gas exchange outlet of the media reservoir. GFP and mCherry images were acquired sequentially. Autofocus was set on the mCherry signal. For the 6-channelled flow chambers (µ-Slide VI 0.4-ibiTreat, *Ibidi*, *Planegg / Martinsried*, *Germany*), in all 5 channels, 7 positions were recorded (Sup. Fig. 7). For the gradient channel (Sup. p.11–14), six positions which correspond to shear stress magnitudes of 2, 5, 8, 11, 13 and 16 dyne/cm^2^ were recorded (Sup. Fig. 8) using 3 × 4 tile scans.

For the 6-well plates, twenty-four well glass-bottomed plates were used with three wells of untreated and three other wells with HUVECs stimulated with TNF-α.

### Image quantification of immunohistochemistry

Wide-field images were imported into MATLAB (*MathWorks*, *MA*, *USA*) using Bio-Formats^39^. The nucleic acid stain was made binary to obtain the nuclei coordinates (Sup. Fig. 9). Background in the NF-κB images was removed with a threshold defined by a sample region with no cells. Based on the obtained coordinates, the nuclear and cytoplasmic intensities per image were quantified. The sum of the nuclei and cytoplasmic intensity was normalised by the sum of the pixels. Further, the intensity of nuclei and cytoplasm were normalized by the intensity of untreated cells. Mean and standard deviation of intensity ratio of experiments and control were calculated with formulas given in the Sup. Material (Sup Eqs 1 and 2).

### Single cell tracking and intensity quantification

Live-cell images were loaded as described for IHC. The raw H2B-Cherry and GFP-RelA images were corrected for background^40^. The background removal process is described in more detail in the Sup. Material (Sup. p.5–7). A software was developing to track the position of each nucleus based on coordinates and area during the time lapse (Sup. p.8). The intensities of each tracked cell were then quantified and normalized by the cell’s time average intensity (Sup. p.8–10). Time average intensity was chosen as significant signals would deviate from the mean.

### Mouse studies

All methods and experimental protocols were approved and carried out according to rules set out by the Imperial College London. Apolipoprotein E-deficient (ApoE^−/−^) mice were instrumented with a blood flow-modifying cuff around the left carotid artery as described before^41^. Mice were imaged *in vivo* using micro-CT (n = 7; isotropic resolution of 39.6 µm) 7 days after cuff placement when new flow profiles have fully developed to assess the effect of imposed flow profiles on the biology of the vessel wall. At each scan time, blood flow velocity measurements were also acquired at the inlet of both carotid arteries using Doppler Ultrasound. Computational fluid dynamics (CFD) was then performed on all 14 scanned vessels (including the left and right carotid arteries within each mouse) to compute wall shear stress.

The expression levels of specific proteins (p65, CD68) were assessed in endothelial cells at regions upstream of the cuff (low shear stress region), regions inside the cuff (high shear region) and regions distal of the cuff (disturbed flow region) of murine carotid arteries by *en face* staining.

### Patient selection

All research was performed in accordance with institutional guidelines and regulations following the recommendations of Helsinki Declaration of theWorld Medical Association. Written informed consent regarding the use of clinical information and biological samples was obtained from all subjects in the context of the @neurIST study protocol. Authorization of the Commission cantonale d’éthique de la recherche de Genève (CCER; original authorization number 07–056; amendment number PB_2018-00073) was obtained. The @neurIST study is a project that collects information and biological samples on patients diagnosed with intracranial aneurysms. The recruitment of patient is consecutive and prospective in Geneva University Hospital since November 1st 2006. Patients incidentally diagnosed with intracranial aneurysms are evaluated by a team of specialists and according to the estimated risk of aneurysm rupture either followed up by regular non-invasive cerebrovascular imaging or further investigated by performing an angiographic study including high resolution 3D digital rotational angiography (Allura Xper FD20, Philips Healthcare, Netherland) (3D-DRA). 3D-DRA is used to generate a model of the patient specific cerebrovascular geometry (Philips Healthcare TM Xtravision, Netherland) and perform computational flow dynamics simulations. On the basis of the clinical and imaging information the team of specialists selects the optimal intervention plan that could be intravascular aneurysm coiling, stenting or microsurgical clipping or a mix of methods. Approximately 30% of all patients treated for an incidental intracranial aneurysm have their aneurysm clipped. Aneurysm dome can only be obtained in the context of aneurysm clipping.

### Aneurysm dome resection

Resection of the aneurysm dome after clipping of the lesion neck is part of the standard surgical procedure initially described by Yasargil^42^ to inspect that no small daughter vessels is accidentally occluded, and that no aneurysm wall “dog-ear” are left. Each patient is sedated and monitored by a dedicated anesthesiology team and neurophysiologist and the aneurysm dome is exposed according to standard micro-neurosurgical approaches. Once the aneurysm dome has been dissected from the surrounding structures, one or multiple microsurgical clips (L-Aneurysm-Clip, Peter Lasic GmbH, Tuttlingen, Germany or Sugita clip, Mizuho Medical Co., Ltd., Tokyo, Japan) are applied on the parent vessel neck to reconstruct the tubular geometry of the vessel or vessel bifurcation. The tight occlusion of the aneurysm neck is proofed by puncture of the dome, lumen emptying by suction and observation of an eventual refilling. A micro-suture is stitched to the aneurysm dome for later specimen orientation. A video or photographs are recorded from the operative microscope to document the aneurysm dome orientation *in situ* and the localization of the micro-suture stitch. The aneurysm wall is cut with surgical micro scissors leaving a 0.5 mm cuff of tissue that will be coagulated to avoid any bleeding or slipping of the clip. The aneurysm dome is then transferred to a sterile tube containing normal saline at room temperature. The aneurysm dome is immediately handled by the laboratory personnel according to the aneurysm dome study protocol.

### Human intracranial aneurysm samples

The samples were immediately rinsed in PBS and fixed 20 minutes with 4% PFA at room temperature. After fixation, all samples were pinned out and flattened for *en face* staining, then permeabilized with 0.2% TritonX-100 (in PBS) for 1 hour, charges were neutralized with 0.5 M NH_4_Cl (in PBS) for 15 min and samples were blocked with 2% BSA (in PBS) for 30 minutes. NFκB p65 antibody (Santa Cruz; sc-109) was diluted 1/100 in blocking solution and incubated on the specimen overnight at 4 °C. Secondary goat anti-rabbit antibodies (Alexa Fluor 488 fluorochrome-conjugated) were used for signal detection. Cytoplasm and nucleic acids were counterstained with 0.003% Evans Blue (Sigma) and 1/20000 DAPI (Invitrogen), respectively. All regions containing endothelium were sampled. Pictures were taken with a LSM800 Airyscan confocal microscope and images have been processed using the software ZEN2.3 (Zeiss).

### Aneurysm computational fluid dynamics

Three-dimensional rotational angiography was segmented in OsiriX (*OsiriX v9*.*0*.*1*, *Freeware*) and post-processed with re-meshing and Gaussian smoothing in Blender (﻿*Stichting Blender Foundation*, *Amsterdam*, *The Netherlands*) and MeshLab (*MeshLAb v1*.*3*.*0*, *Freeware*). The cerebral vasculature was trimmed at a relatively straight portion of the parent vessel to form an inlet plane, approximately five vessel diameters distance distal to the aneurysm. The same procedure was used to trim the vasculature proximal to the aneurysms and form outlet planes. Any side branches in the remaining vasculature were assumed to transport negligible flow proportions and were closed.

A number of assumptions were made to run CFD simulations on the cerebral vasculature segment including assuming rigid vessel walls, a blood density of 1000 kgm^−3^, and Newtonian flow with a constant dynamic viscosity of 0.004 Pas. The geometries were discretised in CFD-VisCART (*ESI Group*, *Paris*) where an unstructured projected single domain con-conforming mesh was used. Mesh independence of velocity and WSS distributions to within 1% was assumed at a mesh fineness previously investigated^43^ and resulted in a simulation minimum mesh size of 2,070,000 cells across the geometries. Population-averaged flow rates were assumed for each vessel segment and inlet boundary conditions applied as a Poiseuille (parabolic) profile (see Supplementary Material p50–53 for more details)^44^ Constant-pressure outlet boundary conditions were imposed assuming symmetrical flow impedances distal to the aneurysm in cases with multiple outlets. In cases where multiple inlets were present, parent vessel flow rates were apportioned using area power law as proposed by Cebral *et al*.^45^.

## Supplementary information

supplementary info

## References

[1] Yurdagul A, Finney AC, Woolard MD, Orr AW (2016). The arterial microenvironment: the where and why of atherosclerosis. Biochem J.

[2] Andreou I (2015). “How do we prevent the vulnerable atherosclerotic plaque from rupturing? Insights from *in vivo* assessments of plaque, vascular remodeling, and local endothelial shear stress”. J Cardiovasc Pharmacol Ther.

[3] Xiao L, Liu Y, Wang N (2014). New paradigms in inflammatory signaling in vascular endothelial cells. Am J Physiol Heart Circ Physiol.

[4] Dabek J, Kulach A, Gasior Z (2010). Nuclear factor kappa-light-chain-enhancer of activated B cells (NF-kappaB): a new potential therapeutic target in atherosclerosis?. Pharmacol Rep.

[5] Robbesyn F, Salvayre R, Negre-Salvayre A (2004). Dual role of oxidized LDL on the NF-kappaB signaling pathway. Free Radic Res.

[6] Diagbouga, M. R., Morel, S., Bijlenga, P. A. P. & Kwak, B. Role of hemodynamics in initiation/growth of intracranial aneurysms, *European Journal of Clinical Investigation* (2018). 10.1111/eci.12992 29962043

[7] Kwak BR (2014). Biomechanical factors in atherosclerosis: mechanisms and clinical implications†.

[8] Pedrigi RM (2017). Disturbed Cyclical Stretch of Endothelial Cells Promotes Nuclear Expression of the Pro-Atherogenic Transcription Factor NF-kappaB. Ann Biomed Eng.

[9] Chlupac J (2014). The gene expression of human endothelial cells is modulated by subendothelial extracellular matrix proteins: short-term response to laminar shear stress. Tissue Eng Part A.

[10] Feaver RE, Gelfand BD, Blackman BR (2013). Human haemodynamic frequency harmonics regulate the inflammatory phenotype of vascular endothelial cells. Nat Commun.

[11] Cuhlmann S (2011). Disturbed blood flow induces RelA expression via c-Jun N-terminal kinase 1: a novel mode of NF-kappaB regulation that promotes arterial inflammation. Circ Res.

[12] Ganguli A (2005). Distinct NF-kappaB regulation by shear stress through Ras-dependent IkappaBalpha oscillations: real-time analysis of flow-mediated activation in live cells. Circ Res.

[13] Tzima E (2002). Activation of Rac1 by shear stress in endothelial cells mediates both cytoskeletal reorganization and effects on gene expression. Embo J.

[14] Nagel T, Resnick N, Dewey CFJr, Gimbrone MA (1999). Vascular endothelial cells respond to spatial gradients in fluid shear stress by enhanced activation of transcription factors, (in eng). Arterioscler Thromb Vasc Biol.

[15] Chien S, Li S, Shyy YJ (1998). Effects of mechanical forces on signal transduction and gene expression in endothelial cells. Hypertension.

[16] Zambrano, S., Bianchi, M. & Agresti, A. High-Throughput Analysis of NF-kappa B Dynamics in Single Cells Reveals Basal Nuclear Localization of NF-kappa B and Spontaneous Activation of Oscillations, (in English), *Plos One*, Article 9, no. 3, MAR 4, Art no. ARTN e90104 (2014). 10.1371/journal.pone.0090104 PMC3942427 24595030

[17] Turner, D. *et al*. Physiological levels of TNF alpha stimulation induce stochastic dynamics of NF-kappa B responses in single living cells, (in English), *Journal of Cell Science*, Article 123, 16, pp. 2834–2843, (2010). 10.1242/jcs.069641 PMC2915884 20663918

[18] Lee, T. *et al*. A Noisy Paracrine Signal Determines the Cellular NF-kappa B Response to Lipopolysaccharide, (in English), *Science Signaling*, Article 2, 93, OCT 20 2009, Art ARTN ra65 (2009). 10.1126/scisignal.2000599 PMC2778577 19843957

[19] Tay, S. *et al*. Single-cell NF-kappa B dynamics reveal digital activation and analogue information processing, (in English), *Nature*, Article 466, 7303, 267-U149, (2010). 10.1038/nature09145 PMC3105528 20581820

[20] Baeyens N (2014). Syndecan 4 is required for endothelial alignment in flow and atheroprotective signaling. Proc Natl Acad Sci USA.

[21] Boon RA (2010). KLF2-induced actin shear fibers control both alignment to flow and JNK signaling in vascular endothelium. Blood.

[22] Kadohama T (2006). p38 Mitogen-activated protein kinase activation in endothelial cell is implicated in cell alignment and elongation induced by fluid shear stress. Endothelium.

[23] Zhang Y (2010). Enhanced external counterpulsation attenuates atherosclerosis progression through modulation of proinflammatory signal pathway. Arterioscler Thromb Vasc Biol.

[24] Mohan S (2007). Low shear stress preferentially enhances IKK activity through selective sources of ROS for persistent activation of NF-kappaB in endothelial cells, (in eng). Am J Physiol Cell Physiol.

[25] Cheng C (2008). Rapamycin modulates the eNOS vs. shear stress relationship. Cardiovasc Res.

[26] Won, D. *et al*. Relative reduction of endothelial nitric-oxide synthase expression and transcription in atherosclerosis-prone regions of the mouse aorta and in an *in Vitro* model of disturbed flow, (in English), *American Journal of Pathology*, Article 171, 5, 1691–1704, (2007). 10.2353/ajpath.2007.060860 PMC2043529 17982133

[27] Seneviratne, A. N. *et al*. Low shear stress induces M1 macrophage polarization in murine thin-cap atherosclerotic plaques, *J Mol Cell Cardiol*, 89, Pt B, 168–72, (2015). 10.1016/j.yjmcc.2015.10.034 26523517

[28] Z. Mohri *et al*. Elevated uptake of plasma macromolecules by regions of arterial wall predisposed to plaque instability in a mouse model, *PLoS One*, 9 12, e115728, (2014). 10.1371/journal.pone.0115728 PMC4274101 25531765

[29] da Silva RF (2008). Role of arginase pathway in response to shear stress: new potential therapeutic targets for atherosclerosis?. European Journal of Clinical Investigation.

[30] Cheng C (2007). Large variations in absolute wall shear stress levels within one species and between species. Atherosclerosis.

[31] Krams R (2006). Shear stress is associated with markers of plaque vulnerability and MMP-9 activity. EuroIntervention: journal of EuroPCR in collaboration with the Working Group on Interventional Cardiology of the European Society of Cardiology.

[32] Carlier SG (2003). Augmentation of wall shear stress inhibits neointimal hyperplasia after stent implantation: inhibition through reduction of inflammation?, (in eng). Circulation.

[33] Mohan, S., Mohan, N. & Sprague, E. Differential activation of NF-kappa B in human aortic endothelial cells conditioned to specific flow environments,” (in English), *American Journal of Physiology-Cell Physiology*, Article 273, 2, C572–C578, (1997). 10.1152/ajpcell.1997.273.2.C572 9277354

[34] Hay D (2003). Activation of NF-kappa B nuclear transcription factor by flow in human endothelial cells,” (in English). Biochimica Et Biophysica Acta-Molecular Cell Research,.

[35] Wang Y (2009). Shear Stress Regulates the Flk-1/Cbl/PI3K/NF-kappa B Pathway Via Actin and Tyrosine Kinases, (in English), *Cellular and Molecular*. Bioengineering, Article.

[36] Zambrano S, Bianchi ME, Agresti A (2014). A simple model of NF-kappaB dynamics reproduces experimental observations. J Theor Biol.

[37] Kis, Z. *et al*. Development of a synthetic gene network to modulate gene expression by mechanical forces, *Scientific Reports*, Article 6, p. 29643, 07/12/online (2016). 10.1038/srep29643 PMC4940741 27404994

[38] Dolan JM, Meng H, Sim FJ, Kolega J (2013). Differential gene expression by endothelial cells under positive and negative streamwise gradients of high wall shear stress. American Journal of Physiology-Cell Physiology.

[39] Openmicroscopy, “BioFormats”, ed., http://www.openmicroscopy.org/.

[40] Schwarzfischer, M., Marr, C., Krumsiek, J. & Theis, F. J. Efficient fluorescence image normalization for time lapse movies, ed. Proc. Microscopic Image Analysis with Applications in Biology (2011).

[41] Cheng C (2006). Atherosclerotic lesion size and vulnerability are determined by patterns of fluid shear stress. Circulation.

[42] Yasargil, M. G. *Microneurosurgery: Clinical Considerations*, *Surgery of the Intracranial Aneurysms and Results* (1984).

[43] Peach T, Spranger K, Ventikos Y (2017). Virtual flow-diverter treatment planning: The effect of device placement on bifurcation aneurysm haemodynamics,”. Proceedings of the Institution of Mechanical Engineers, Part H: Journal of Engineering in Medicine.

[44] Zarrinkoob L (2015). Blood Flow Distribution in Cerebral Arteries. Journal of Cerebral Blood Flow & Metabolism.

[45] Cebral JR, Castro MA, Putman CM, Alperin Flow-area N (2008). relationship in internal carotid and vertebral arteries. Physiological measurement.

