# Supplementary material for: Disturbed flow induces a sustained, stochastic NF-κB activation which may support intracranial aneurysm growth in vivo
Source: Sci Rep. 2019 Mar 18;9:4738. doi: 10.1038/s41598-019-40959-y (PMC6426999; doi:10.1038/s41598-019-40959-y)
Supplement: Supplementary file 1 — supplementary info [file 41598_2019_40959_MOESM1_ESM.docx]

# Disturbed flow induces a sustained, stochastic NF-κB activation which may support intracranial aneurysm growth *in vivo*

Daniel C. Baeriswyl^1^, Ioanna Prionisti^2^, Tom Peach^1^, Grigoris Tsolkas^2^, Kok Yean Chooi^2,3^, John Vardakis^1^, Sandrine Morel^4^, Mannekomba R Diagbouga^4^, Philippe Bijlenga^6^, Simon Cuhlmann^2^, Paul Evans^2,5^, Brenda R Kwak^4^, Yiannis Ventikos^1^ and Rob Krams^2,3^

^1^ Dept. Mechanical Engineering, University College London, ^2^ Dept. Bioengineering, Imperial College London, ^3^ SEMS, Queen Mary London, ^4^ Dept Pathology and Immunology, University of Geneva, Switzerland, ^5^ dept. Medicine, University of Sheffield, and ^6^ Dept. Clinical Neurosciences, Geneva University Hospitals, Switzerland.

**Supplementary Material**

Contents

[GFP-RelA and H2B-mCherry Plasmids	3](#_Toc534297947)

[Transfection protocol	4](#_Toc534297948)

[Perfusion system:	5](#_Toc534297949)

[Image quantification of immunohistochemistry	7](#_Toc534297950)

[Single cell tracking and Intensity quantification	8](#_Toc534297951)

[Normalization:	12](#_Toc534297952)

[Design and evaluation of a flow chamber with a linear increase in shear stress.	13](#_Toc534297953)

[Time laps of nuclear translocation of GFP-RelA in HUVECs exposed to 10ng/mL TNF-alpha	17](#_Toc534297954)

[Time laps of nuclear translocation of GFP-RelA and H2B-mCherry in HUVECs exposed to 10ng/mL TNF-alpha:	18](#_Toc534297955)

[Kmean clustering single cell measurements of different conditions:	19](#_Toc534297956)

[Modelling the NF-kB pathway	20](#_Toc534297957)

[Receptors activation	21](#_Toc534297958)

[TNF-alpha stimulation	22](#_Toc534297959)

[IκBα-NFκB signaling pathway	22](#_Toc534297960)

[Cell population read outs	27](#_Toc534297961)

[Modelling shear stress stimulation	32](#_Toc534297962)

[Model assumptions of the shear stress induced NF-κB activation	32](#_Toc534297963)

[Shear stress dependent stimulation of the NF-κB pathway	32](#_Toc534297964)

[Initial, early, mid and late activation groups	35](#_Toc534297965)

[Gradient channel - Geometry and meshing	41](#_Toc534297966)

[Backward facing step channel – Geometry and meshing	42](#_Toc534297967)

[Governing equations, solver and boundary conditions for CFD simulations (Gradient channel, backward facing step channel and aneurysms)	43](#_Toc534297968)

[Boundary conditions gradient channel	43](#_Toc534297969)

[Grid independence gradient channel	44](#_Toc534297970)

[Gradient channel shear stress profile	45](#_Toc534297971)

[Streamlines within the gradient channel	45](#_Toc534297972)

[Boundary conditions backward facing step channel	47](#_Toc534297973)

[Grid independence backward facing step channel	47](#_Toc534297974)

[Shear stress profile at the bottom wall of a backward facing step channel	47](#_Toc534297975)

[Aneurysms: Computational fluid dynamics setup	49](#_Toc534297976)

[Aneurysms: Parent vessel flow rates	49](#_Toc534297977)

[Aneurysms: Inlet velocity profiles	49](#_Toc534297978)

[Aneurysms: Inlet flow split boundary conditions	50](#_Toc534297979)

[Animals and surgery	53](#_Toc534297980)

[In vivo µCT imaging	53](#_Toc534297981)

[Doppler velocity measurements	54](#_Toc534297982)

[Segmentation and 3-D reconstruction	55](#_Toc534297983)

[Carotid artery meshing and computational fluid dynamics	56](#_Toc534297984)

# GFP-RelA and H2B-mCherry Plasmids

Figure 1: Full sequence map of GFP-RelA. RelA sequence was inserted in the ORF frame 2 ^1^. GFP-RelA was a gift from Warner Greene (Addgene plasmid # 23255)

Figure 2:Full sequence map of H2B-mCherry ^1^. H2B-mCherry was a gift from Robert Benezra (Addgene plasmid # 20972

# Transfection protocol

Confluent HUVECs in a tissue culture flask were trypsinized and incubated for 2 minutes at 37°C. Supernatant was added to neutralize the trypsin and the solution was centrifuged at 280 RCF for 5 minutes at 37°C. The supernatant was removed, and the cell pellet was used for transfection. A NEON Transfection System (Thermo Fisher Scientific, MA, USA) was used for electroporation of cells. A mixture of 500,000 cells, 60ul R Buffer, 20uL H2B-mCherry and 20ul GFPRelA was prepared for each transfection. The most efficient and successfully transfection setting was found with 2 pulses of 1,000 Volts, with a duration of 20us per pulse. After transfection, the cells were immediately collected and diluted in Endothelial Growth Media. The mixture was then centrifuged, the remaining buffer and plasmid solutions was removed, and fresh growth media was added. The final solution of transfected HUVECs was used in well plates or flow experiments.

The HUVECs of passage 3 were transfected with GFP-RelA and H2B-mCherry and cultured for 4 days. In Figure 3 confocal images of the HUVECs expressing GFP-RelA and H2B-mCherry under static conditions are shown. The transfection efficiency was around 60-80%. The GFP-RelA intensity was calculated in the merged GFP-RelA +H2B-mCherry channel, which allowed us to separate between nuclear and cytoplasmic GFP-RelA concentration.

|   A |   B |   C |
| --- | --- | --- |
| Figure 3:Confocal image of HUVECs expressing GFP-RelA (A) and H2B-mCherry (B) under static conditions. Both channels are merged (C) and thus the nuclear concentration of GFP-RelA can be calculated. | | |

# Perfusion system:

|  | |  |
| --- | --- | --- |
| Figure 4: Schematic diagram of the perfusion system containing a peristaltic pump, two dampers, a pressure transducer, an ultrasound probe and a flow chamber. All parts were connected using silicon tubes and connectors. The flow chamber was placed directly under a microscope when performing live-cell imaging experiments. For immunohistochemistry, the experiment was performed in an incubator. | |  |
|  |   B | |
| Figure 5: A: Flow rate measurements at different pump speeds. B: Average flow rates as a funciton of pump speed with a indicated regression line.  A | | |
|   A |   B | |
| Figure 6: Flow rate (A) and pressure (B) measurements of the perfusion system without and with 2 dampers at a pump speed of 10x. | | |

Figure 7: Recording positions during live-cell imaging experiment with a 6-channelled Ibidi slide. Positions are placed in the developed flow area

A

B

Figure 8: Positions at different shear stress magnitudes along the gradient channel (A). 3x4 tiles scan of GFP-RelA signal (B)

# Image quantification of immunohistochemistry

Wide-field images were imported into MATLAB (*MathWorks, MA, USA*) using Bio-Formats ^2^. These were then analysed by calculating the cytoplasmic and nuclear intensity. To achieve this, the nuclear image was made binary (Figure 9.A) to obtain nuclei coordinates. Subsequently, the nuclear and cytoplasmic intensity of p65 (Figure 9.B-D) was calculated based on the obtained coordinates. The nuclear and cytoplasmic extraction of p65 are shown in Figure 9.B and Figure 9.C. The background was manually corrected for by applying a threshold that was defined by empty spots (areas were no cells were grown). The sum of the nuclei and cytoplasmic intensity was normalised by the sum of the pixels. Once the nuclei and cytoplasm were calculated the mean and standard deviation of the ratio between experimental and static conditions were calculated using the following formulas:

|  | $E\left( \frac{R}{S} \right)\approx\frac{ER}{ES}-\frac{Cov\left( R,S \right)}{E^{2}S}+\frac{Var\left( S \right)ER}{E^{3}S}$ | ( 1 ) |
| --- | --- | --- |
|  | $Var\left( \frac{R}{S} \right)=\frac{{\mu^{2}}_{R}}{{\mu^{2}}_{S}}\left[ \frac{{\sigma^{2}}_{R}}{{\mu^{2}}_{R}}-\frac{Cov\left( R,S \right)}{\mu_{R}\mu_{S}}+\frac{{\sigma^{2}}_{S}}{{\mu^{2}}_{S}} \right]$ | ( 2 ) |

Where *ER* is the expected value of *R*, and *ES* the expected value of *S*, $E\left( \frac{R}{S} \right)$ is the expected value of the ratio *R* to *S*. (Expected value is the mean). $\mu$ is the mean of *E* or *S*, $\sigma$ is the standard deviation of *E* or *S*. *Var* is the variance, and finally the standard deviation is the root of the variance. In our case, *R* was the mean of experiment sample and *S* the mean of the static conditions sample. The ratios of mean and standard deviation were calculated for the cytoplasm and nuclear concentration for p65.

|   A  B  C |  |
| --- | --- |
| Figure 9: *The binary nuclei image (A) and the nuclei (B) and cytoplasm (C) extraction of the p65 image.* | |

# Single cell tracking and Intensity quantification

Live-cell images series were loaded into MATLAB (*MathWorks, MA, USA*) using Bio-Formats ^2^. The tracking algorithm is depicted schematically in diagram of Figure 10.

Figure 10: Schematic diagram of single cell tracking algorithm and nuclear GFP-RelA intensity calculation. First, the raw image of H2B-mCherry and GFP-RelA were processed by enhancing the contrast, correcting non-uniform illumination and removing noise with a median filter. Then the H2B-mCherry image was made binary and the nuclei were numbered. The numbered nuclei were tracked throughout all time frames. The coordinates from the tracked nuclei were used to calculate the nuclear GFP-RelA intensity in the corrected GFP-RelA image. Cells nuclear GFP-RelA intensity were plotted, and later normalised by either time average or earliest common time point. The population mean of the normalised nuclear GFPRelA intensity including standard deviation was plotted as the final result.

First, the raw images of H2B-mCherry and GFP-RelA were corrected for the

Background ^3^ as shown in Figure 11. The correction involved the following steps: The contrast of the raw image was enhanced (Figure 11.B), then the non-uniform background (Figure 11.C) and the regression (Figure 11.D) were calculated. Subsequently, the final image (Figure 11.E) was calculated using Equation ( 3 ).

|  |  | | ( 3 ) |  |
| --- | --- | --- | --- | --- |
|   A | | | | |
|   B  D  C | | | | |
|   E  E | | Figure 11: A: The raw wide-field image. B: Enhanced contrast. C: Non-uniform illumination background. D: The regression of the background. E: The final image after enhancing, removing the background and divide by the regression. | | |

The background was calculated by dividing the image into 32 equal squares. Each square was averaged and blurred with its surrounding to create a smooth

background. The regression was calculated by dividing the background image using the mean of the background image. Regression aimed to overcome photo bleaching in time. The H2B-mCherry image was made binary as shown in Figure 12. Each white dot represents a nucleus.

Software was developed to compare the position of each nucleus between each time step. The smallest difference in position and the similarity of area determined which cell, in the next time frame, was the cell from the previous time frame. The tracking of cells under two experimental conditions is shown in Figure 13. The time series of nuclei were superimposed, and a thin pink line indicates the pathways of the nuclei. Not all cells could be tracked. Cells that went into apoptosis and cells that divided were rejected. Cells that suddenly appeared from outside the frame after the first-time step were also ignored. Approximate 30% of the cells were rejected.

|  | |
| --- | --- |
| Figure 12: The H2B-mCherry image made binary, representing the cells’ nucleus. | |
|   A |   B |
| Figure 13: The pathways (pink line) of tracked nuclei when stimulated with TNF-α (A) and exposed to high shear stress (B) | |

Once cells were tracked in their pathway, the GFP-RelA intensity at the coordinates of the nucleus per time step was calculated for each cell as the mean pixel intensity:

|  | $I_{n}(t)[a.u]=\frac{\sum_{i}^{P} G_{i,n}(t)}{P_{n}(t)}$ | ( 4 ) |
| --- | --- | --- |

Where $G_{i, n}(t)$ is the nuclear GFP-RelA intensity per i-th pixel for the n-th nuclei at time point *t*. $P_{n}(t)$ is the number of pixel per nuclei at time point *t.* $I_{n}\left( t \right)$ is the mean pixel nuclear GFP-RelA intensity per cell at time point *t*. We will always refer to this quantity as the nuclear GFP-RelA intensity.

|   A |   B |
| --- | --- |
|  |   C |
| Figure 14: The intensity of 200 cells when stimulated with TNF-α (A), under static conditions (B) and exposed to high shear stress (C). | |

Additionally, a very weak smoothing was applied to remove major noise (Savitzy-Golay, degree = 0.1). However, the filter was very weak (almost not noticeable) to avoid filtering any possible oscillation of the signal. The strengthening of the filter was chosen such that the TNF-α measurement with the wide-field still peaked at 30 minutes. A too strong filter resulted in offsetting or cutting out the major peak when stimulated with TNF-α. This was due to a large recording time interval. Due to the large time interval (dT =10-15 min) a nuclear GFP-RelA peak of 30 minutes was captured with approximated 3 points. A too strong filter cuts out any peak occurring within these 3 measurement points. Any common moving averaging filter is not suitable for our signals as it cuts off/shifts major peaks. Savitzy-Golay is a polynomial filter which is suitable for filtering noise of signals with large frequency spans ^4^. In Figure 14, the nuclear GFP-RelA intensity of 200 cells for three different experiments (TNF-α, static conditions and high shear stress) are shown. One can see distinctive features between the experiments.

However, the single cell measurements include also a lot of noise and a huge cell-to-cell variability. Hence, proper normalization of the data was necessary to draw meaningful conclusion.

# Normalization:

For each cell, the GFP-RelA intensity at the coordinates of the nucleus per time step was calculated cell as the mean pixel intensity

|  | $I_{n}(t)[a.u]=\frac{\sum_{i}^{P} G_{i,n}(t)}{P_{n}(t)}$ | ( 5 ) |
| --- | --- | --- |

Where $G_{i, n}(t)$ is the nuclear GFP-RelA intensity per i-th pixel for the n-th nuclei at time point *t*. $P_{n}(t)$ is the number of pixel per nuclei at time point *t.* $I_{n}\left( t \right)$ is the mean pixel nuclear GFP-RelA intensity per cell per time point.

We normalize the GFP-RelA intensity by the time average

|  | $I_{n}^{M}=\frac{I_{n}\left( t \right)}{\left\langle I_{n}\left( T_{start}<t<T_{end} \right) \right\rangle}$ | ( 6 ) |
| --- | --- | --- |

$T_{start}$ is the start time that all positions have in common. Due to camera speed, the last position of the gradient channel was recorded for the first time at t=15 minutes. Therefore, we define $T_{start}=15 min$. $T_{end}$ is the end time that all positions have in common, which is $T_{end}=360 min.$

# Design and evaluation of a flow chamber with a linear increase in shear stress.

Gradient channel design:

The gradient channel was designed to have a shear stress gradient from 2 to 16 dyne/cm^2^. The height for both end points was calculated with

|  | $Ht=\sqrt{\frac{6uQ}{\tau\omega}}$ | ( 7 ) |
| --- | --- | --- |

With Q = 2.5e-7m^3^/s (15mL/min), μ=0.78e^-3^ Pa s (DMEM at 37ºC) and H=4cm. For a shear stress of 2 dyne/cm^2 this resulted in an approximate height of ℎk=400 m and for 16 dyne/cm^2^ a height of ℎl=1400 m. To calculate a linear increasing shear stress, the following formula given by Usami et al. [ref] was used

|  | $\tau=\frac{6\mu Q (1-\frac{z}{L})}{h^{2}\omega}$ | ( 8 ) |
| --- | --- | --- |

The channel height as a function of z derivate from Equation (1) and (2) is given as:

|  | $h\left( z \right)=\sqrt{\frac{h_{l}^{2}}{L-z}}L$ | ( 9 ) |
| --- | --- | --- |

Additionally, the entrance length was calculated to ensure an established flow before and after the gradient section. This was calculated with the formula for the entrance length in laminar flows:

|  | $L=0.05Red_{h}$ | ( 10 ) |
| --- | --- | --- |

|  | $Re=\frac{\rho uh(z)}{\mu}$ | ( 11 ) |
| --- | --- | --- |
|  | $d_{h}=\frac{2h\left( z \right)w}{h\left( z \right)+w}$ | ( 12 ) |

The entrance length with the velocities expected at ℎ_k_ and ℎ_l_ would be 5.6 mm and 0.91 mm, respectively. To increase the presence of stationary parabolic velocity profiles, the entrance and exit section were both set to be 10mm long. Additionally, the gradient channel was perfused from the large cross section to the smaller one, leading to a linear increase in shear stress in the flow direction. The final three-dimensional gradient channel with the above calculated dimension is shown in Figure 15.

The flow characteristics of the gradient channel were studied with CFD (more detailed at p.44-49) using CFD-ACE *(ESI Group, Paris, France).* In Figure 16A, the shear stress profile of the cross-section along the bottom wall of the gradient channel is shown. It shows a gradually increasing shear stress from 15mm until 52mm. Coloured squares indicate the positions at which the live-cell imaging was recorded. The live-cell imaging was recorded at a shear stress of 2, 5, 8, 11, 13 and 16 dyne/cm^2. The shear stress profiles of the recording position (red, blue, green, purple, cyan and yellow) of the cross-section in the Y-direction are shown in Figure 16B. It shows the increase in shear stress magnitude, and that at 1 cm away from both the side walls (1 and 2.7cm) the shear stress profiles were uniform. Therefore, the recording positions were positioned in the middle of the Y-cross section to ensure a uniform shear stress profile.

In Figure 16C, the streamlines along the whole channel are shown. The flow enters the gradient channel from the first pillar without major disturbances. Only a small recirculation zone existed at the far bottom left of the pillar.

Figure 15: 3-dimensional gradient channel domain for flow validation with computational fluid dynamics.

|   a |   b |
| --- | --- |
|   c | |
| Figure 16: Shear stress profile (A) along the channel at the bottom wall (z=0cm) at the middle of the channel (x=2cm). The positions for live-cell imaging are indicated with color-coded square symbols. The shear stress profile along the Y-axis (B) at the bottom wall (z=0cm) for the live-cell imaging positions. Shear stress profiles are colour-coded with the position indicated in A. The streamline profile of the gradient channel (C). | |

The mould of the final geometry was designed in SolidWorks *(Dassault Systèmes, Vélizy-Villacoublay, France)* as shown in Figure 17. The gradient channel was milled without the inlet pillars. The inlet pillars were added separately by first drilling two holes and then hammering in two pillars with the appropriate diameter. The gradient channel mould was manufactured using a DATRON C5 - 5 AXIS machine (Datron, Milford, US), available at the Hamlyn Centre at Imperial College London, that has an accuracy of 2 m. As a material, we used stainless steel.

Once the mould was manufactured, an additional case made of PVC was fitted around the model to create a basin/mould cast for the liquid PDMS. The actual channel is then manufactured by pouring liquid PDMS into the mould cast. We used Sylgard® 184 Silicone Elastomer Kit (Dow Corning, Auburn, US) with a mixture of 10:1. The PDMS is then cured at 60°C for 1.5 hours, after which it was carefully removed from the mould. We used Menzel Gläser (Thermo Scientific, Waltham, US) for the bottom of the channel as a growth surface for the cells. To bind the PDMS gradient channel to glass, we treated the surface with a Plasma Prep (Gala Instrument, Manchester, UK) for 30 seconds at 0.4mbar. The final gradient channel is shown in Figure 18. Before using the gradient channel for tissue culture application, the gradient channel was cleaned by flushing with ethanol and distilled water and then autoclaved for sterility. Prior to seeding cells, the inner channel bottom surface was coated with 0.01% Poly-L-lysine solution (Sigma Aldrich, Mo, USA) and 0.1% gelation solution by incubated for 1-2 hours at 37°C.

Figure 17: The gradient channel mould geometry manufactured with a micro milling machine.

Figure 18: The final gradient channel after the PDMS and glass are bonded together.

| **GFP-RelA** | | | |
| --- | --- | --- | --- |
|   **t = 0 min** |   **t = 12 min** |   **t = 35min** |   **t = 60 min** |
|   **t = 96 min** |   **t = 126 min** |   **t = 242 min** |   **t = 360 min** |
| Figure 19*:HUVECs expressing GFP-RelA after exposure to 10 ng/mL TNF-α at different time points. High nuclear GFP-RelA intensity in the nucleus is observed at 38, 60, 96 and 126 minutes. At 242 and 360 minutes, nuclear GFP-RelA concentration is low and almost as at 0 minutes. This movie like time frame demonstrates live the translocation of GFP-RelA. Variations between cells can be observed. Imaged with confocal.* | | | |

# Time laps of nuclear translocation of GFP-RelA in HUVECs exposed to 10ng/mL TNF-alpha

| **GFP-RelA and H2B-mCherry** | | | |
| --- | --- | --- | --- |
|   **t = 0 min** |   **t = 12 min** |   **t = 38 min** |   **t = 60 min** |
|   **t = 96 min** |   **t = 126 min** |   **t = 242 min** |   **t = 360 min** |
| *Figure 20: HUVECs expressing GFP-RelA and H2B-mCherry after exposure to 10 ng/mL TNF-α at different time points. High nuclear GFP-RelA intensity in the nucleus is observed at 38, 60, 96 and 126 minutes. At 242 and 360 minutes, nuclear GFP-RelA concentration is low and almost as at 0 minutes. This movie like time frame demonstrates live the translocation of GFP-RelA in and out of the nucleus. Variations between cells can be observed. Imaged with confocal.* | | | |

# Time laps of nuclear translocation of GFP-RelA and H2B-mCherry in HUVECs exposed to 10ng/mL TNF-alpha:

# Kmean clustering single cell measurements of different conditions:

|   c  a |  |
| --- | --- |
|  |   d  b |
|   e |   f |
|   g |   h |
| Figure 21: Cluster map and the mean of each cluster of the nuclear GFP-RelA intensity normalized by time average of HUVECs exposed to 10ng/mL TNF-alpha **(a,b)**, untreated **(c,d)**, uniform low shear stress **(e,f)** and uniform high shear stress **(g,h)**. | |

# Modelling the NF-kB pathway

The overarching goal of the modelling effort undertaken in this dissertation was to simulate the NF-κB pathway of an entire cell population in response to a stimulus. The cell population model is schematically shown in Figure 22.

Due to the naturally occurring heterogeneity in a cell population ^5^, differences in cell shape, surface receptors, cell orientation and cell life cycles may be present. We have developed a methodology based on a Monte Carlo technique that addresses heterogeneity and thus captures each individual cell response. For the heterogeneity, only extrinsic noise that influences each cell’s individual activation and reaction rates of certain biochemical processes was considered ^6^ ^7^.

C

D

E

B

A

Figure 22: Schematic of the cell population model. A) Cells are stimulated by TNF-α or shear stress. B) The stimulus activates surface receptors of each cell. Due to differences of cells in a population, the number of activated receptors per cell varies. The number of activated receptors is a random number of a Gaussian normal distribution. The intensity of the stimulus defines the mean of the normal distribution. High intensity defines a high mean, while low intensity implies a low mean. Based on a random seed, we select from the stimulus-depended distribution the number of initially activated receptors per cell. C) The number of activated receptors possesses a temporal b ehaviour with initial delay, random in- or activation, fast and slow decay. A threshold a defines if a cell is activated or not. In other words, if a cell has less than a receptors activated, it is inactive. If more than a receptors are activated, the cell is active and the NF-κB pathway is triggered. D) The NF-κB signalling pathway with reaction rate φ which is a function of the activated receptors. E) The nuclear NF-κB concentration of a cell population of 200 cells stimulated with 10 ng/mL TNF-α.

We hypothesised that all cells have the same amount of surface receptors, *M*. A stimulus activates the surface receptors. However, the number of activated receptors is defined by the intensity of the stimulus. This variation of activated receptors per cell of a population is represented by a Gaussian normal distribution. The use of a Gaussian normal distribution is aimed at representing cell heterogeneity. Heterogeneity is defined herein solely by the number of activated surface receptors, which is per se a simplification of the total external noise such as cell size, state of cell and measurable properties.

The stimulus intensity defines the mean of such a normal distribution. For example, if cells are exposed to high TNF-α concentration then the mean of the receptor activation Gaussian normal distribution will be high. If the TNF-α concentration is low, the mean of the normal distribution will be low. Random numbers are then selected from the normal distribution to define the initially activated receptors of each cell. The activated receptors have a temporal behaviour including initial delay, random in- or activation, fast and slow decay.

In addition, a threshold function was defined, such that a cell only gets activated if more surface receptors are activated than a pre-defined threshold *a*. If less than *a* surface receptors are activated, the cell remains inactive. Active defines that the NF-κB pathway is triggered with the activation rate$\phi\left( t \right)$ larger than zero. ($\phi\left( t \right)$ is a function of activated surface receptors). Inactive cells imply the pathway is not initiated and the activation rate $\phi\left( t \right)$ is equal to zero. The NF-κB pathway was then calculated for each cell with each cells individual activation rate *φ*. An example of the nuclear NF-κB concentration for 200 cells stimulated with 10 ng/mL TNF-α is shown in Figure 13.E.

# Receptors activation

The temporal change in activated receptors of each cell *i* was defined as:

| $r_{i}\left( t \right)=tanh\left( \frac{1}{T_{Delay}\cdot w_{Delay}} \right)\left( 1-k_{C}\cdot tanh\left( \frac{1}{T_{FDecay}\cdot w_{FDecay}} \right) \right)\left( X_{i}+k_{L}\cdot w_{L}-e^{\frac{t}{T_{SDecay}}} \right)$ | ( 2 ) |
| --- | --- |

The receptor activation equation approximates the binding of free TNF-α molecules to TNFR1 ^8,9^.

- Initial delay: $\tanh\left( \frac{1}{T_{Delay}\cdot w_{Delay}} \right)$ , with delay time $T_{Delay}$ and a random number $w_{Delay}\in\{0, \ldots,1\}$
- Fast decay: $1-k_{c}\cdot\tanh\left( \frac{1}{T_{FDecay}\cdot w_{FDelay}} \right)$*,* with decay time $T_{Fdecay}$ , $k_{c}$ is a constant and a random number $w_{Fdecay}\in\{0, \ldots,1\}$. The fast decay was included due to suggestion by Cheong *et al.* ^10^ that IKK activation has a quick decay after initial stimulation.
- Initially activated receptors: The number $X_{i}$ is randomly picked for each *i-th* cell from the Gaussian normal distribution $P_{i}\left( b_{i},\mu\left( t \right) \right)$with a mean as a function of stimuli intensity $\mu(t)$. The Gaussian normal distribution and $X_{i}$ are given a

|  | $P_{i}\left( b_{i},\mu\left( t \right) \right)=\frac{1}{\sigma\sqrt{2\pi}}e^{\frac{-\left( b_{i}-\mu\left( t \right) \right)^{2}}{2\sigma}}$ | ( 3 ) |
| --- | --- | --- |
|  | $b_{i}=\left\{ b_{i}\mathfrak{\in R,-}\frac{M}{2}\leq b_{i}\leq\frac{3M}{2} \right\}$ | ( 4 ) |
|  | $X_{i}=\left\{ \begin{matrix} X_{i}=0 & b_{i}<0 \\ X_{i}\in P_{i} & 0\leq b_{i}\leq M \\ X_{i}=M & b_{i}>0 \end{matrix} \right.$ | ( 5 ) |

$P_{i}\left( b_{i},\mu(t) \right)$ is the receptor activation probability, $\sigma$ the standard deviation, and *M* is the total number of receptors of each cell. $b_{i}$ is the span of the normal distribution and $X_{i}$ is the randomly chosen number of activated receptors. Note: If $X_{i}$ is chosen where $b_{i}$ is smaller than zero then $X_{i}$ is zero. If $X_{i}$ is chosen where $b_{i}$ is greater M then $X_{i}$ is equal to *M*.

- Random walk: A random number picked from $w_{L}\in\{-1,0,1\}$
- Slow decay: ${-e}^{\frac{t}{T_{SDecay}}}$ is a natural decay function with slow decay time $T_{Sdecay}$.

# TNF-alpha stimulation

The stimuli intensity $\mu(t)$ when stimulated with TNF-α is given as

|  | $\mu\left( t \right)=\alpha\cdot{TNF}^{2}+\beta\cdot TNF+\gamma,$ | ( 6 ) |
| --- | --- | --- |

where *TNF* is the TNF-α concentration. The parameters used for this equation were $\alpha=-10.9635, \beta=267.1747$ and $\gamma=130.2175$.

Stimulation by flow is described later.

# IκBα-NFκB signaling pathway

The NF-κB pathway is described schematically in Figure 23. IKK has three states: Neutral, active and inactive. Active IKK phosphorylates IκBα, of the IκBα-NFκB complex, and causes ubiquitination of IκBα. This unbinds NF-κB, which can then freely travel into a nucleus. Nuclear translocated NF-κB binds to DNA and transcribes new IκBα and A20. The transcribed IκBα and A20 can then synthesise to form proteins in the cytoplasm. Subsequently, the newly synthesised cytoplasmic IκBα proteins bind again to form NF-κB and inhibit its function and mobility. Newly synthesised A20 causes the inactivation of IKK. Therefore, the NF-κB pathway is a negative feedback loop ^11,12^.

In this work, the mathematical model of the NF-κB pathway described by Lipniacki *at al.* ^12^ was implemented in the cell population model. Moreover, the model was extended by defining a new activation scale $\phi_{i}\left( t \right)$ for each cell

|  | $\phi_{i}\left( t \right)=\left\{ \begin{matrix} \frac{r_{i}\left( t \right)-a}{M-a}, & r_{i}\left( t \right)>a \\ 0, & r_{i}\left( t \right)\leq a \end{matrix} \right.$ | ( 7 ) |
| --- | --- | --- |

Where *a* is a threshold. In cases were the number of activated receptors $r_{i}\left( t \right)$ is lower than the threshold $a$, the activation scale is zero. If the activated receptors $r_{i}\left( t \right)$ are above the threshold $a$, the activation scale is the rate of activated receptors over the maximum number of receptors ***M***. Therefore, if the number of activated receptors is above $a$, the cell is activated. If the number is below *a*, the cell is inactive.

Using the activation scale, the reaction rates in the IκBα-NF-κB signaling pathway were proportioned. First, the IKK activation rate was scaled with the above defined scale $\phi_{i}\left( t \right)$. This approach was adopted based on the suggestion by Cheong *et al.* ^10^ that the IKK activation has a temporal dynamic with a sharp decaying curve. Moreover, Turner *et al.* suggested modification of the IKK activation with a decay function that includes a random walk. In this work, the activation rate of IKK was scaled proportional to the number of activated surface receptors as described in Equations ( 2 ) and ( 7 ). Therefore, the activation rate of IKK was affected by the stimuli intensity, fast and slow decays, a random walk and a threshold cut-off.

Figure 23: Schematic of the NF-κB pathway with a cell. The reaction rates that are influenced by activated receptors $\boldsymbol{\phi}_{\boldsymbol{i}}\left( \boldsymbol{t} \right)$, the IKK activation and the nuclear import of NF-κB are highlighted in red.

Second, we have scaled the nuclear import of NF-κB proportional to the number of activated receptors. This allowed the establishment of a nuclear NF-κB peak that is proportional to the stimuli. The nuclear import rate was scaled based on the assumption that the nuclear transport of NF-κB facilitated by Importin-α3 and Importin-β4 is a TNF-α influenced mechanism ^13,14^. Other studies have used stochastic models to reduce the nuclear NF-κB amplitude as the probability of dissection and association decreases with stimuli intensity ^15^. However, in this work the aim was to simplify the model to a deterministic model by using extrinsic noise only. In scaling the reaction rate of nuclear import of NF-κB, the nuclear NF-κB peaks could be reduced proportional to the stimuli. Including this feature, allowed for qualitative reproduction of the live-cell measurements of transfected HUVECs exposed to TNF-α and shear stress.

The receptor dependent reaction rates of the NF-κB model are:

- The IKK activation rate caused by TNF-α

|  | $k_{1}=0.5163\cdot\phi_{i}\left( t \right)$ | ( 8 ) |
| --- | --- | --- |

- Nuclear import rate of NF-κB

|  | $i_{1}=0.0026\cdot\phi_{i}\left( t \right)$ | ( 9 ) |
| --- | --- | --- |

All reaction rate constants were based on the work of Lipniacki *et al.* ^12^. Moreover, these were extended with the activation scale $\phi_{i}\left( t \right)$ and fitted to nuclear GFP-RelA measurements presented in Chapter 3. The full set of all biochemical reactions of the IkBa-NFkB signalling pathway ^12^ are:

- The neutral state of IKK:

|  | $\frac{d}{dt}{IKK}_{n}\left( t \right)=k_{prod}-k_{deg}\cdot{IKK}_{n}\left( t \right)-k_{1}\left( t \right)\cdot{IKK}_{n}\left( t \right)$ | ( 10 ) |
| --- | --- | --- |

- The activation of IKK:

|  | $\frac{d}{dt}IKK_{a}\left( t \right)=k_{1}\left( t \right)\cdot IKK_{n}\left( t \right)-k_{3}\cdot IKK_{a}\left( t \right)-k_{2}\left( t \right)\cdot IKK_{a}\left( t \right)\cdot A_{20}\left( t \right)-k_{deg}\cdot IKK_{a}\left( t \right)-a_{2}\cdot IKK_{a}\left( t \right)\cdot I\kappa B\alpha\left( t \right)+t_{1}\left( t \right)\cdot IKK_{a}I\kappa B\alpha-a_{3}\left( t \right)\cdot IKK_{a}\left( t \right)\cdot I\kappa B\alpha NF\kappa B\left( t \right)+t_{2}\left( t \right)\cdot IKK_{a}I\kappa B\alpha NF\kappa B\left( t \right)$ | ( 11 ) |
| --- | --- | --- |

- The inactivation of IKK:

|  | $\frac{d}{dt}{IKK}_{i}\left( t \right)=k_{3}\left( t \right)\cdot IKK_{a}\left( t \right)+k_{2}\left( t \right)\cdot IKK_{a}\left( t \right)\cdot A_{20}\left( t \right)-k_{deg}\cdot IKK_{i}\left( t \right)$ | ( 12 ) |
| --- | --- | --- |

- Active IKK and IκBα complex that degrades IκBα:

|  | $\frac{d}{dt}IKK_{a}I\kappa B\alpha\left( t \right)= a_{2}\left( t \right)\cdot IKK_{a}\left( t \right)\cdot I\kappa B\alpha\left( t \right)\cdot-t_{1}\left( t \right)\cdot IKK_{a}I\kappa B\alpha\left( t \right)$ | ( 13 ) |
| --- | --- | --- |

- Active IKK association with the IκBα-NFκB complex:

| $\frac{d}{dt}IKK_{a}I\kappa B\alpha NF\kappa B\left( t \right)= a_{3}\left( t \right)\cdot IKK_{a}\left( t \right)\cdot I\kappa B\alpha NF\kappa B\left( t \right)-t_{2}\left( t \right)\cdot IKK_{a}I\kappa B\alpha NF\kappa B\left( t \right)$ | ( 14 ) |
| --- | --- |

- The unbound cytoplasmic NFκB:

|  | $\frac{d}{dt}\mathrm{NF}\kappa B=c_{6a}\cdot I\kappa B\alpha NF\kappa B\left( t \right)-a_{1}\cdot\mathrm{NF}\kappa B\left( t \right)\cdot I\kappa B\alpha\left( t \right)+t_{2}\left( t \right)\cdot IKK_{a}I\kappa B\alpha NF\kappa B\left( t \right)-i_{1}\left( t \right)\cdot\mathrm{NF}\kappa B\left( t \right)$ | ( 15 ) |
| --- | --- | --- |

- Unbound nuclear NFkB:

|  | $\frac{d}{dt}\mathrm{NF}\kappa B_{n}= i_{1}\left( t \right)\cdot k_{v}\cdot\mathrm{NF}\kappa B\left( t \right)-a_{1}\cdot I\kappa B\alpha_{n}\left( t \right)\cdot\mathrm{NF}\kappa B_{n}\left( t \right)$ | ( 16 ) |
| --- | --- | --- |

- Inhibitor A20 protein:

|  | $\frac{d}{dt}A_{20}\left( t \right)= c_{4}\cdot A_{20mRNA}\left( t \right)-c_{5}\cdot A_{20}\left( t \right)$ | ( 17 ) |
| --- | --- | --- |

- A20 mRNA:

|  | $\frac{d}{dt}A_{20mRNA}\left( t \right)= c_{2}+c_{1}\cdot\mathrm{NF}\kappa B_{n}\left( t \right)-c_{3}\cdot A_{20mRNA}\left( t \right)$ | ( 18 ) |
| --- | --- | --- |

- Unbound IkBa protein:

|  | $\frac{d}{dt}I\kappa B\alpha\left( t \right)= -a_{2}\left( t \right)\cdot IKK_{a}\left( t \right)\cdot I\kappa B\alpha\left( t \right)-a_{1}\left( t \right)\cdot I\kappa B\alpha\left( t \right)\cdot\mathrm{NF}\kappa B\left( t \right)+c_{4a}\cdot I\kappa B\alpha_{mRNA}\left( t \right)-c_{5a}\cdot I\kappa B\alpha\left( t \right)-i_{1a}\cdot I\kappa B\alpha\left( t \right)+e_{1a}\cdot I\kappa B\alpha_{n}\left( t \right)$ | ( 19 ) |
| --- | --- | --- |

- Unbound nuclear IkBa protein:

|  | $\frac{d}{dt}I\kappa B\alpha_{n}\left( t \right)=-a_{1}\left( t \right)\cdot I\kappa B\alpha_{n}\left( t \right)\cdot\mathrm{NF}\kappa B_{n}\left( t \right)+i_{1a}\cdot k_{v}\cdot I\kappa B\alpha\left( t \right)-e_{1a}\cdot k_{v}\cdot I\kappa B\alpha_{n}\left( t \right)$ | ( 20 ) |
| --- | --- | --- |

- IkBa mRNA:

|  | $\frac{d}{dt}I\kappa B\alpha_{mRNA}\left( t \right)=c_{2a}+c_{1a}\cdot\mathrm{NF}\kappa B_{n}\left( t \right)-c_{3a}\cdot I\kappa B\alpha_{\mathrm{mRNA}}\left( t \right)$ | ( 21 ) |
| --- | --- | --- |

- Cytoplasmic IkBaNFkB complex:

|  | $\frac{d}{dt}I\kappa B\alpha NF\kappa B\left( t \right)=a_{1}\left( t \right)\cdot I\kappa B\alpha\left( t \right)\cdot\mathrm{NF}\kappa B\left( t \right)\cdot-c_{6a}\cdot I\kappa B\alpha NF\kappa B\left( t \right)-a_{3}\left( t \right)\cdot IKK_{a}\left( t \right)\cdot I\kappa B\alpha NF\kappa B\left( t \right)+e_{2a}\cdot I\kappa B\alpha_{n}\mathrm{NF}\kappa B_{n}\left( t \right)$ | ( 22 ) |
| --- | --- | --- |

- Nuclear IkBaNFkB complex:

|  | $\frac{d}{dt}I\kappa B\alpha_{n}\mathrm{NF}\kappa B_{n}\left( t \right)=a_{1}\left( t \right)\cdot I\kappa B\alpha_{n}\left( t \right)\cdot\mathrm{NF}\kappa B_{n}\left( t \right)-e_{2a}\cdot k_{v}\cdot I\kappa B\alpha_{n}\mathrm{NF}\kappa B_{n}\left( t \right)$  Table 1: Parameter with * were fitted, for the others the references are indicated. | | | | | | | | ( 23 ) | |
| --- | --- | --- | --- | --- | --- | --- | --- | --- | --- | --- |
| **Reaction rate** | | $K_{v}$ ^12^ | $C_{1}$ ^12^ | $C_{2}$ ^12^ | $C_{3}$ ^12^ | $C_{4}$ ^12^ | $K_{prod}$* | $K_{deg}$* | |  |
| **value** | | 5 | 5e-7 | 0 | 0.004 | 0.5 | 2.62e-5 | 1.2e-4 | |  |
| **Reaction rate** | | $A_{1}$* | $C_{1a}$* | $C_{2a}$* | $C_{3a}$* | $C_{4a}$* | $C_{5a}$* | $C_{6a}$* | |  |
| **value** | | 0.5026 | 3.23e-7 | 0 | 4..7e-4 | 0.4814 | 1.14e-4 | 1.77e-5 | |  |
| **Reaction rate** | | $E_{2a}$* | $i_{1a}$* | $E_{1a}$* | $k_{2}$* | $a_{2}$* | $a_{3}$* | $t_{1}$ ^12^ | |  |
| **value** | | 0.0105 | 0.001 | 4.9e-4 | 0.0918 | 0.2089 | 1.152 | 0.1 | |  |
| **Parameter** | | $t_{2}$* | $k_{3}$* | $T_{delay}$ | $k_{C}$ | $T_{FDecay}$ | $k_{L}$ | $T_{SDecay}$ | |  |
| **value** | | 0.1064 | 0.0016 | 7 | 0.65 | 46.125 | 50 | 307 | |  |
|  | | | | | | | | | |  |

All constant parameters are provided in Table 1. The parameters were fitted using *fminsearch* in *Matlab* (*MathWorks, Natick, Massachusetts, US*), employing a simplex search method by Lagarias *et al.* ^16^. Within the search method, the minimum error between model and experiment was found using a simple error calculation:

|  | $\varepsilon=\sqrt[2]{S_{Model}-S_{Experiment}}$ | ( 24 ) |
| --- | --- | --- |

Arbitrary units were used for the model and the experiments, and the curves were fitted until they reached a qualitative similar shape.

# Cell population read outs

The results of the shear induced NF-κB population model are presented with the following quantities extracted from the NF-κB pathway model depicted in section 0:

- The total nuclear NF-κB concentration normalised by time average:

|  | $\mathrm{NF}\kappa B_{Nuc}\left( t \right)=\frac{\mathrm{NF}\kappa B\left( t \right)+I\kappa B\alpha NF\kappa B_{n}\left( t \right)+E}{\left\langle\mathrm{NF}\kappa B(t)+I\kappa B\alpha NF\kappa B_{n}\left( t \right)+E \right\rangle}$ | ( 25 ) |
| --- | --- | --- |

Where *E*=0.035 is an included measurement noise to allow normalisation of inactive cells and mimic experimental conditions.

- The total cytoplasmic IκBα concentration:

|  | ${I\kappa B\alpha}_{Cyto}\left( t \right)= IKK_{a}I\kappa B\alpha\left( t \right)+IKK_{a}I\kappa B\alpha NF\kappa B\left( t \right)+I\kappa B\alpha\left( t \right)+I\kappa B\alpha NF\kappa B\left( t \right)$ | ( 26 ) |
| --- | --- | --- |

- And the active IKK concentration:

|  | $IKK_{Activated}\left( t \right)= IKK_{a}+IKK_{a}I\kappa B\alpha\left( t \right)+IKK_{a}I\kappa B\alpha NF\kappa B\left( t \right)$ | ( 27 ) |
| --- | --- | --- |

The nuclear NF-κB concentration was normalised using the time average (for comparison with the live-cell imaging experiments), while active IKK and IκBα were giving in arbitrary concentration units.

| *Simulation* | *Experiment* | | |
| --- | --- | --- | --- |
|   A |   E | | |
|   B |   F | | |
|   C |   G | | |
|   D |   H | | |
| Figure 24: Comparison of cell population simulation (A, B, C, D) and experiment (E – confocal, F – wide-field, G – confocal, H – wide-field). The cluster maps of nuclear NF-κB concentration when stimulated with 100 ng/mL TNF-α (A, D), 10 ng/mL TNF-α (B,E), 1 ng/mL TNF-α (C,F) and under static conditions (D,H). | | | |
| *Simulation* | | *Live-cell Imaging* |  |
|   A | |   E |  |
|   B | |   F |  |
|   C | |   G |  |
|   H  D | |  |  |
| Figure 25: Comparison of cell population simulations (A, B, C, D) and experiment (E – confocal, F – wide-field, G – confocal, H – wide-field). The mean of the nuclear NF-κB concentration and GFP-RelA intensity when stimulated with 100 ng/mL (A, D), 10 ng/mL (B, E), and 1 ng/mL TNF-α (C, F) and under static conditions (D, H). | | |  |

| Simulation | Simulation | Immunohistochemistry |
| --- | --- | --- |
| A | E | Not performed |
| B | I  F |  |
| C | G | J |
| D | H | Not performed |
| Figure 26: Cell population simulation (200 cells) when stimulated with 100 ng/mL (A, E), 10 ng/mL (B, F, I), 1 ng/mL TNF-α (C, G, J) and under static conditions (D, H). The left column (A-D) shows the temporal profile of active IKK of all cells grouped and mapped. The middle column (E-H) shows the total population average of active IKK with its standard deviation. The right column (I and J) shows immuhistochemistry of nuclear and cytoplasmic phorphosrylated IKKα/β (active IKK). | | |

| Simulation | Simulation | Immunohistochemistry |
| --- | --- | --- |
| A | E | Not performed |
| B | F  I |  |
| G  C | J |  |
| D  H |  | Not performed |
| Figure 27: Cell population simulation (200 cells) when stimulated with 100 ng/mL (A, E), 10 ng/mL (B, F), 1 ng/mL TNF-α (C, G) and under static conditions (D, H). The left column (A-D) shows the temporal profile of active IKK of all cells grouped and mapped. The right column (E-H) shows the total population average of active IKK with its standard deviation. | | |

# Modelling shear stress stimulation

A modelling effort to predict the moderate changes observed in nuclear GFP-RelA intensity in HUVECs exposed to a shear stress gradient is presented. The model is based on identified Kmeans groups found in the single cell measurements (normalised by time average) in the gradient channel. Five groups were identified: Initial activation, early activation, mid activation, late activation and inactive cells. The grouping was considered as an approximation of how nuclear translocation of GFP-RelA in single cells exposed to shear stress may occur. As will be demonstrated, reproduction of such groups was achieved reasonable well. The prediction of nuclear NF-κB in cells exposed to a gradient channel was qualitatively in good agreement with experimental observations. However, this modelling effort was based on visual inspected grouping of activation groups. Therefore, the presented link between shear stress and NF-κB activation provides a suggestion of how such complex systems could be predicted.

# Model assumptions of the shear stress induced NF-κB activation

- Receptor based IKK activation mimics shear induced NF-κB activation via Flk-1, integrins, AKT and PI3K ^17^.
- Randomness in receptor activation mimics cell heterogeneity including factors such as: cell size, cell state and orientation
- Model parameters are assumed to be independent of stimuli and are based on TNF-α observations.
- Activation groups were identified with Kmeans by visual inspection
- Kmeans were performed with the nuclear GFP-RelA intensity normalised by the time average
- Relationships between activation groups and shear stress were fitted based on experimental measurements within a gradient channel (Chapter 4) to a linear regression (initial, mid, late) or second order polynomial regression (early activation group).

# Shear stress dependent stimulation of the NF-κB pathway

In cases were flow was the stimulus of receptor activation, it was assumed that flow activated through PECAM-1, VEGF2, integrin and Flk-1 via AKT and PI3K the IKK, and the NF-κB pathway ^17^ ^18^. This complex signalling pathway was simplified by applying the same receptor activation profile as defined for the TNF-α. It was assumed that flow activates a certain number of receptors that trigger the NF-κB pathway in a similar manner to TNF-α. Using this approach, the link between flow and activation of NF-κB was shortened and simplified.

The shear induced NF-κB stimulated model parameters were based on the model fitting to TNF-α. This assumed that the NF-κB pathway has a universal dynamic (independent of stimuli). The intensity of the flow stimuli was accordingly tailored, and each activation group experienced a stimulus comparable with 1-5 ng/mL TNF-α.

Five groups that experience different time dependent stimuli intensities $\mu(t)$were defined. The number of cells per groups was shear stress dependent.

- Case 1 - Inactive cells:

|  | $\mu_{R}\left( t \right)=27, R=1,2,3, \ldots, R_{max}\left( \tau\right)$ | ( 28 ) |
| --- | --- | --- |

Cells remain inactive (but can experience random activation) throughout the experiment. The total number of inactive cells is $R_{max}\left( \tau\right)$.

- Case 2: Initial activation group:

|  | $\mu_{A}\left( t=0 \right)=1090, A=1,2,3, \ldots, A_{max}\left( \tau\right)$ | ( 29 ) |
| --- | --- | --- |

Cells experience activation at 0 minutes. The total number of initial activation cells is $A_{max}\left( \tau\right)$.

- Case 2: Early activation group:

|  | $\mu_{B}\left( t \right)=\left\{ \begin{matrix} 788, & 15+\xi_{B}<t<360 \\ 27, & t<15+\xi_{B} \end{matrix} \right.$ | ( 30 ) |
| --- | --- | --- |
|  | $B=1,2,3, \ldots, B_{max}\left( \tau\right)$ | ( 31 ) |

$\xi_{B}$ is a random number picked from $\xi_{B}\epsilon\left\{ 0,\ldots, 60 \right\}$. Cells experience random activation between 15 and 75 minutes. The total number of early activation cells is $B_{max}\left( \tau\right)$.

- Case 3: Mid activation group:

|  | $\mu_{C}\left( t \right)=\left\{ \begin{matrix} 677, & 125+\xi_{C}<t<360 \\ 27, & t<125+\xi_{C} \end{matrix} \right.$ | ( 32 ) |
| --- | --- | --- |
|  | $C=1,2,3, \ldots, C_{max}\left( \tau\right)$ | ( 33 ) |

$\xi_{C}$ is a random number picked from $\xi_{C}\epsilon\left\{ 0,\ldots90 \right\}$. Cells experience random activation between 125 and 215 minutes. The total number of mid activation cells is $C_{max}\left( \tau\right)$.

- Case 4: Late activation group:

|  | $\mu_{D}\left( t \right)=\left\{ \begin{matrix} 778, & 245+\xi_{D}<t<360 \\ 27, & t<245+\xi_{D} \end{matrix} \right.$ | ( 34 ) |
| --- | --- | --- |
|  | $D=1,2,3, \ldots, D_{max}\left( \tau\right)$ | ( 35 ) |

$\xi_{D}$ is a random number picked from $\xi_{D}\epsilon\left\{ 0,\ldots90 \right\}$. Cells experience random activation between 245 and 335 minutes. The total number of early activation cells is $D_{max}\left( \tau\right)$.

The total number of cells in each group sums the total number of cells of the population. Thus, with *E* as the total number of cells in the population, we can write:

|  | $\frac{1}{E}\left( A_{max}\left( \tau\right)+ B_{max}\left( \tau\right)+ C_{max}\left( \tau\right)+D_{max}\left( \tau\right)+ R_{max}\left( \tau\right) \right)=1$ | ( 36 ) |
| --- | --- | --- |

The number of cells of each activation group was shear dependent. The percentage of cell population ($\frac{A_{max}}{E},\frac{B_{max}}{E},\frac{C_{max}}{E},\frac{D_{max}}{E})$ vs. shear stress is depicted in Figure 28. The shear dependency of the groups was based on observation made with the gradient channel experiments.

Each group’s cell number can be written as an empirical function of shear stress:

|  | | $\frac{A_{max}\left( \tau\right)}{E}=-0.11935\cdot\tau+9.3632$ | ( 37 ) | |
| --- | --- | --- | --- | --- |
|  | | $\frac{B_{max}\left( \tau\right)}{E}=0.0821\cdot\tau^{2}-0.5485\cdot\tau+13.7780$ | ( 38 ) | |
|  | | $\frac{C_{max}\left( \tau\right)}{E}=-0.8862\cdot\tau+26.8841$ | ( 39 ) | |
|  | | $\frac{D_{max}\left( \tau\right)}{E}=-1.2120\cdot\tau+27.1389$ | ( 40 ) | |
|  |  | | |  |
|  | Figure 28: The shear stress dependency of the activation groups based on the measurement of the nuclear GFP-RelA intensity of cells exposed to a shear stress gradient. | | |  |

The time dependent stimuli intensity of each cell was then fed into a receptor activation equation (Eq.( 2 )) and the NF-κB pathway, as described in section 0, was calculated for each cell.

# Initial, early, mid and late activation groups

Based on experimental observations of nuclear translocation of GFP-RelA in a shear stress gradient the flow triggers for each of the four groups at four different time ranges could be defined. The time ranges are: Initial activation (0 min), early activation range (15 – 75 min), mid activation range (110 – 150 min) and late activation range (275 – 335 min).

During each time span a certain percent of cells were activated. Inactive cells also experience random activation. Based on experimental observations in a shear stress gradient (2-16 dyne/cm^2), a mean percentage for each group was defined for 1500 cells within the shear stress gradient as shown in Table 2.

The temporal changes of activated receptors for 1500 cells based on the activation groups defined in Table 6 are depicted in Figure 29.A. The time range for the four activation groups is indicated: initial (green) early (red), mid (magenta) and late (blue). A constant high activation of receptors throughout the 360 minutes was observed.

In Figure 29.B, the number of activated cells in a cell population exposed to a shear stress gradient of 2 – 16 dyne/cm^2 for 360 minutes using the mean percentages from Table 2 are shown. The number of activated cells increases linearly until approximately 190 minutes. From 190 minutes onwards, the number of activated cells remains around 600.

|  | **Time span [min]** | **% of total population** |
| --- | --- | --- |
| *Initial Activation Range:* | 0 | $\frac{A_{max}}{E} = 0.10$ |
| *Early Activation Range:* | 15 – 75 | $\frac{A_{max}}{E} = 0.2$ |
| *Mid activation range:* | 110 – 190 | $\frac{B_{max}}{E} = 0.2$ |
| *Late activation range:* | 235 – 325 | $\frac{C_{max}}{E} = 0.2$ |
| *Inactive with random activation:* | 0 – 360 | $\frac{R_{max}}{E} = 0.3$ |
| Table 2: Time range and percentage of total population of activation groups | | |
| A | | |
| B | | |
| Figure 29: A: The activation profile of 1500 cells in a shear stress gradient. Cells were activated at 3 different time ranges: early (15 – 75 min, red), mid (120 - 180 min, blue) and late (250 – 330 min, green) activation. The threshold is indicated at 200 cells. B: The number of activated cells with indicated activation ranges. | | |

| *Simulation* | *Experiment* |  |
| --- | --- | --- |
| A | C |  |
| B | D |  |
| Figure 30: A: Cluster map of the predicted temporal nuclear NF-κB concentration (A) and measured nuclear GFP-RelA intensity (C) of a cell population exposed to a shear stress gradient of 2-16 dyne/cm^2. Means of the predicted temporal nuclear NF-κB concentration (B) and measured nuclear GFP-RelA intensity (C) of each cluster group. The total mean is indicated in red. The peaks of each group and the percentages of the total population are indicated. | | |
| *Simulation* | *Simulation* | |
| A | C | |
| B | D | |
| Figure 31: Cluster map of the temporal activated IKK(A) and IκBα (C) concentration of 1500 cells exposed to a shear stress gradient (2 – 16 dyne/cm^2). Means of the temporal activated IKK(B) and IκBα (D) concentration of each clustered group and the total mean (red). In addition, the peaks of each group and the percentages of the total population are indicated. *Simulation* \| \| *Experiment* \|; A \| \| B \|; \| Figure 32: A: Cell population simulation results: The mean of nuclear NF-κB concentration of 300 cells at shear stress 1-16 dyne/cm^2 displayed as a contour map. B: experimental results: The mean nuclear GFP-RelA intensity measured at 2, 5, 8, 11, 13 and 16 dyne/cm^2 (values in-between were interpolated). \| \|; *Simulation* \| \| *Simulation* \|; A \| \| B \|; \| Figure 33: Cell population results of the mean of activated IKK (A) and cytoplasmic IκBα (B) at shear stress magnitudes of 1-16 dyne/cm^2. \| \| | | |

|  | *Simulation* | | *Experiment –*  *Gradient Channel* | *Experiment –*  *Straight Channel* |
| --- | --- | --- | --- | --- |
| *Low shear stress* | |  |  |  |
| *High shear stress* | |  |  |  |
| Figure 34: Comparison of low and high shear stress results of the cell population simulation, gradient channel and straight channel experiments. | | | | |

|  | *Simulation -* ${IKK}_{a}\left( t \right)$ | *Simulation -* ${I\kappa B\alpha}_{Cyto}\left( t \right)$ |  |
| --- | --- | --- | --- |
| *Low shear stress* |  |  |  |
| *High shear stress* |  |  |  |
| Figure 35: Cell population results for active IKK and cytoplasmic IκBα for low and high shear stress (2 and 16 dyne/cm^2). | | |  |
| A  D  C  B | | | |
| Figure 36: : The shear stress profile along the bottom wall of the step channel (A). The NF-κB (B), active IKK (C) and IκBα (D) concentration throughout the channel at three different time point (90, 180 and 360 minutes). | | | |

# Gradient channel - Geometry and meshing

The geometry and dimensions of the gradient channel fluid domain are shown in Figure 37. The mesh (non-conforming, Cartesian, with several levels of subdivisions) was created with VisCart (*ESI Group, Paris, France)*. A cross section of the mesh domain is shown in Figure 37.D and a magnified view of the beginning of the narrowing cross section in Figure 37.E.

| A | |
| --- | --- |
| B | |
| C | |
| D | |
| Figure 37: The geometry (A) and dimensions (B and C) of the gradient channel fluid domain. A cross-section of the mesh domain (D). Magnified view of the mesh at beginning of the narrowing cross section (E). | E |
|  |  |

# Backward facing step channel – Geometry and meshing

In Figure 38A, the geometry of the backward facing step channel is depicted. In Figure 38.B, the mesh of the corner of the sudden expansion is shown.

The mesh was made very dense at the sharp corner of the expansion to capture the rapid inertia redistribution patterns created by a sudden expansion.

A

B

Figure 38: A: The backward facing step channel geometry with dimensions and characteristic flow areas at the bottom wall. B: The mesh at the corner of the sudden expansion. The corner is indicated in the geometry with a green square

# Governing equations, solver and boundary conditions for CFD simulations (Gradient channel, backward facing step channel and aneurysms)

The fluid flow is governed by the mass conservation (or continuity equation)

|  | $\frac{\partial\rho}{\partial t}+\nabla\cdot\left( \rho\vec{u} \right)=0$ | ( 32 ) |
| --- | --- | --- |

and the Navier-Stokes equation (conservation of momentum)

|  | $\rho\left( \frac{\partial\vec{u}}{\partial t}+\left( \vec{u}\cdot\nabla\right)\vec{u} \right)=-\nabla p+\mu\nabla^{2}\vec{u}$ | ( 33 ) |
| --- | --- | --- |

where $\rho$ is the density, $\vec{u}$ the velocity vector (3 dimensional) and *p* the pressure. To solve the governing equations, we used the commercial software CFD-ACE+ from the ESI Group (*ESI, Paris, France*). CFD-ACE+ is based on the finite volume method.

The finite volume method integrates the governing equation over a control volume (The entire flow domain is split into many control volumes as shown in Figures 37 and 38). A central difference scheme (second order accuracy) was applied for spatial discretization. Additionally, CFD-ACE+ uses a blending for the spatial discretization. Blending includes a certain percentage of upwind discretization to the central difference to enhance stability. In our case, the blending was set to a very low value, 0.1 (10% upwind and 90% central difference). This level of blending reduces numerical instabilities without compromising accuracy^19^. The equation system of the velocity field is solved with a CGS (conjugate gradient squared method) solver using 50 sweeps and a convergence criterion of residual reduction to at least five orders of magnitude^19^. Convergence acceleration is achieved with an AMG (algebraic multigrid) method^19^. As no governing equation is giving for the pressure^19,20^. CFD-ACE+ uses the SIMPLEC (Semi-Implicit Method for Pressure-Linked Equations – Consistent) scheme to formulate an equation for the pressure and to couple it with the velocity (The momentum equations ( 32 ) and ( 33 ) are solved for the velocity components). The maximum system iterations were set to 2000 and the minimum residual to 10^-18.

# Boundary conditions gradient channel

The boundary conditions for the gradient channel were: Inlet velocity *u* = 0.0199m/s, Outlet pressure *p* = 0 N/m^2^ and all remaining walls were non-slip walls. The physical properties of the fluid were set for DMEM +10%FBS at 37˚C with µ=0.000786 Pas and $\rho$=1001.13 kg/m^3^ ^21^.

# Grid independence gradient channel

Grid independence results for the gradient channel is shown in Figure 39. The velocity profile at four important cross-sections, specifically: Inlet, converging cross-section, narrow channel outflow and outflow pillar (identified with A, B, C, and D) are plotted for 3 different grids.

The three different grids possessed 800k, 5 million and 22 million mesh elements. Regions of higher inertia and steep changes such as the primary bend from inlet to narrow channel and the outflow from the narrow channel to the outflow pillar were meshed densely. Regions with less inertia and smoother changes were meshed with a lower density of elements. In Figure 152, the velocity profile at point A and B were considered grid independent for all grids in this study. At cross-section C and D, grid independence was achieved with 5 million elements (Relative error between 22M and 5M was <5%).

The narrow outflow into the pillar created a jet like outflow with higher inertia forces than at other regions within the channel. However, our main interest is the flow section in the narrowing channel, for which grid independent was achieved for all grids considered. We concluded that 5 million mesh elements were sufficient to achieve a grid independent flow simulation.

|  | A  B  C  D | |  |
| --- | --- | --- | --- |
|  | |  |  |
|  | |  |  |
| Figure 39: Grid independence study of the gradient channel at 4 different locations: Inlet (A), convergent channel (B), narrowest cross section outflow (C), and outflow (D). | | | |

# Gradient channel shear stress profile

In Figure 40, the shear stress profile of the cross-section along the bottom wall of the gradient channel is shown. It shows a gradually increasing shear stress from 15mm until 52mm.

Coloured squares indicate the positions at which the live-cell imaging was recorded. The live-cell imaging was recorded at a shear stress of 2, 5, 8, 11, 13 and 16 dyne/cm^2. The shear stress profiles of the recording position (red, blue, green, purple, cyan and yellow) of the cross-section in the Y-direction are shown. It shows the increase in shear stress magnitude, and that at 1 cm away from both the side walls (1 and 2.7cm) the shear stress profiles were uniform. Therefore, the recording positions were positioned in the middle of the Y-cross section to ensure a uniform shear stress profile.

| A | B |
| --- | --- |
| Figure 40: Shear stress profile (A) along the channel at the bottom wall (z=0cm) at the middle of the channel (x=2cm). The positions for live-cell imaging are indicated with color-coded square symbols. The shear stress profile along the Y-axis (B) at the bottom wall (z=0cm) for the live-cell imaging positions. Shear stress profiles are colour-coded with the position indicated in A. | |

# Streamlines within the gradient channel

In Figure 41, streamlines within the gradient channel are shown for different views. Figure 41.A shows the streamlines along the whole channel. The flow enters the gradient channel from the first pillar without major disturbances. Only a small recirculation zone existed at the far bottom left of the pillar (Figure 41.B and Figure 41.C).

The convergence of the channel cross-section increased the flow velocity without causing major unsteadiness; streamlines did not show any secondary vortices. The transition from the narrow cross-section to the out-flow pillar caused a jet that created a swirling motion. The jet and swirling motion are shown in more detail in Figure 41.D and Figure 41.E. The swirl ranges from the bottom to the top of the pillar. However, due to the long (10cm) straight section after the convergent section, the swirl is not affecting the flow in the gradient channel where we performed measurements of cells. Hence, we can regard the swirl as negligible. It is important to guarantee little disturbance within the gradient channel to ensure that cells are only affected by a linear shear stress gradient. Large flow disturbance at the in- and outflow might propagate or penetrate through the channel and may affect the cells’ behaviour and change the conditions they are exposed to.

|  | A  C  B |
| --- | --- |
| D  E |  |
| Figure 41: Streamlines of the gradient channel (A), entrance (B and C) and the outlet (D and E). | |

# Boundary conditions backward facing step channel

The boundary conditions for the step channel were: Inlet velocity U = 0.4m/s, Outlet pressure p = 0 N/m^2^ and all remaining walls were non-slip walls. The physical properties of the fluid were set to µ=0.001 Pas and $\rho$ =1000 kg/m^3^. For the step channel, we assumed water at room temperature to be able to compare and validate the CFD results with experimental measurements as shown later in the results section.

# Grid independence backward facing step channel

For the backward facing step channel (2D simulation), we performed a grid independent analysis with four grids: 50k, 100k, 250k and 1 million mesh elements. Grid independence was achieved with relative coarse grid of 250k elements.

Figure 42: Comparison of reattachment point of the numerical simulation with measurements by Armaly et al. ^22^

One could validate the simulation result with 250k elements against experimental findings from Armaly et al. ^22^ by comparing the comparing the reattachment point (*x* = location with zero velocity, *S* = height of the step) as shown in Figure 42. We found good agreement of the reattachment point for Reynold numbers between 100-300. Above a Reynolds number 300, the resolution was poor, and additionally flow instabilities start to occur which would necessitate transient simulations and/or 3D flow simulations ^23^ being conducted. In this work, it was proposed that a backward facing step channel with a low Reynolds numbers such as 100-300 would be investigated.

# Shear stress profile at the bottom wall of a backward facing step channel

In Figure 43, shear stress profiles at the bottom wall of the backward-facing step channel at different Reynolds numbers (50 – 250) are shown. The bottom wall of the step channel can be divided into four different regions: Stagnation zone, recirculation zone, flow reattachment and developed flow zone.

With an increasing Reynolds number, the shear stress increased at all zones, whereas the increases were largest at the recirculation zone. Our shear profile of interest was found to be at a Reynolds number of 200. At this Re, a shear stress range of 0 to 16 dyne/cm^2 was observed. This range is suitable for the shear induced NF-κB simulation. Measurements were experimentally obtained of the nuclear NF-kB intensity of cells exposed to a similar shear stress range (2-16 dyne/cm^2) in a shear stress gradient channel.

Figure 43: Shear stress profiles of the backward-facing step channel at different Reynolds numbers

# Aneurysms: Computational fluid dynamics setup

CFD simulations were run in the CFD-ACE+ suite (ESI Group) as previously detailed on page 44. Blood flow was modeled as an incompressible fluid with unsteady 3D Navier-Stokes governing equations, with second-order interpolations in space and time, a time-step of 0.001 second, a rigid arterial wall, a fluid density of 1000 kg/m3, and a dynamic viscosity of 0.004 Pa s. We adopted a Newtonian fluid model, assuming that non-Newtonian flow effects are small in both the aneurysm dome and arteries with a diameter of >0.5 mm.

Simulations were run on 32 2.93-GHz cores, with each time-step converging to five orders of magnitude residual reduction in around 50 iterations in a typical solution time of 30 minutes per time-step. A constant heart rate of 75 BPM was assumed and three full cardiac cycles were simulated taking the results of the third cycle in order to reduce the influence of initial transient flow effects.

# Aneurysms: Parent vessel flow rates

The patient-specific cerebral flow rates were not recorded for this cohort of patients. Consequently, average values of both total cerebral blood flow and proportion of total flow in the arterial segment were used to estimate parent vessel flow rates, using values from Zarrinkoob et al^24^.

# Aneurysms: Inlet velocity profiles

The velocity profile of flow at the parent vessels were unknown. Simulating the entire vascular tree proximal to the vessel in question in order to obtain a velocity profile was considered too computationally costly and would be reliant upon inaccurate estimates of the terminating impedances of other elements of the Circle of Willis. Consequently, the sensitivity of aneurysm Wall Shear Stress (WSS) to the choice of an Anterior Cerebral Artery (ACA) inlet velocity profile was briefly investigated.

Two inlet velocity profiles were proposed: (1) a uniform plug profile and (2) a radially symmetric parabolic profile (Poiseuille) typical of pressure-driven flow in a pipe or vessel with low curvature. These velocity distributions are shown below in Figure 44.

Figure 44: two cross sections with the velocity profiles are shown for plug flow or parabolic flow

Next, the wall shear stress magnitude projected on the wall of the vasculature surrounding the aneurysm was plotted below for each inlet velocity profile and compared (Figure 45). As judged from the figure, only small, insignificant difference is seen in aneurysm WSS due to inlet velocity profile. The Poiseuille (parabolic) profile is chosen for improved convergence of the CFD solution and for having a more physiological basis.

Figure 45: WSS projected onto the vessel wall in the vicinity of and including the aneurysm for each inlet velocity profile: plug and Poiseuille

# Aneurysms: Inlet flow split boundary conditions

In geometries with multiple inlets, such as the ACA system, the distribution of overall flow in each vessel is unknown. Relative changes in flow rate between the two branches may dramatically alter the WSS distribution and magnitude in the aneurysm dome. Without MR or similar patient-specific measurements of blood velocity, the L-R distribution ACA flow rates must be inferred.

Two flow distributions are proposed: (1) an even split of flow between left and right ACA (43ml/min in each) and (2) a flow split based on the power-law metric proposed by Cebral et al.^25^ which links cerebral artery lumen area and relative flow rate. The power law-based approach predicted a 62:24 ml/min flow split between left and right ACAs.

Constant pressure outlet boundary conditions were imposed assuming symmetrical flow impedance distal to the ACAs.

Wall shear stress magnitude plots for each flow split are shown in Figure 46 for the aneurysm region. Similar overall WSS patterns are seen between the two flow conditions, however the WSS magnitude in the aneurysm is higher in the case of the 62:24 (power law) flow split.

Figure 46: WSS projected on the wall of the aneurysm after two flow splits (see text for further details)

Additionally, average parent vessel WSS magnitude values are indicated below in Figure 47 for each flow split simulated. The power law flow split results in more even distributions of WSS in the two parent vessels.

Figure 47: A more extensive view of the effect on flow split onto the WSS distribution of the aneurysm and vessels in close proximity of the aneurysm

Flow split by area power law (as proposed by Cebral et.al.^25^) offers more reasonable WSS distributions in parent vasculature. Where multiple parent vessel inlet exist inlet flow rates are apportioned by area power-law for maximum fidelity.

# Animals and surgery

ApoE-/- mice (~10 weeks old) were commercially obtained (The Jackson Laboratory, Bar Harbor, Maine, USA) and housed in the animal unit at the South Kensington campus and Hammersmith Hospital campus, Imperial College London. A colony was maintained, and their offspring were used for all experiments. A maximum of 5 animals were placed in a single cage and were given food and water ad libitum. Animal care and all experimental procedures complied with the Animals (Scientific Procedures) Act 1986 and were approved by the Home Office, UK. Female ApoE-/- mice (20-23 grams in weight) were used for all experiments. Seven mice went through the surgical procedure of placing the flow-modifying cuff on the left carotid arteries, of which 2 were used for imaging and 5 for histology. The cuff is made up of two thermoplastic polyetherketone cylindrical halves with a conical lumen that tapers from 500 µm at the base to 250 µm at the throat of the cuff. Placement of the cuff creates a gradual stenosis in the artery, leading to three distinct regions of wall shear stress; low shear stress upstream of the cuff, high shear stress within the cuff, and low oscillatory shear stress (characterised by vortices) downstream of the cuff, as shown previously by Cheng et al.^26^

All mice were weighed prior to the surgical procedure. Anaesthesia was induced with an intra-peritoneal injection of a solution containing Domitor (Medetomidine, 1mg/ml, Orion Pharma), Hypnovel (Midazolam, 5mg/ml, Roche), Vetergesic (Buprenorphine, 0.1mg/ml, Alstoe Animal Health), and saline in ratios depending on their body weight. They were then placed on a heating pad to maintain their body temperature during the surgical procedure. Fur was removed from the neck region from the mandible to the sternum using a commercially available hair removal cream or by use of a fine electric shaver. Following an incision in the neck region, the subcutaneous tissue was teased open, giving access to the parotid gland. The gland was carefully lifted and rested out of the incision while being continuously hydrated, and the deeply seated left carotid artery and trachea region were exposed by teasing through the tissue. The perivascular cuff (Promolding, Rijswijk, the Netherlands) was then placed around the left common carotid artery (the contralateral right carotid artery is used as the control vessel), held firmly in place by a suture, with care taken to maintain at least a ~1 mm distance from the carotid bifurcation. The parotid gland was gently moved back into its original position before suturing the neck wound. Post-surgery, a subcutaneous injection of Antisedan (Atipamezole, 5mg/ml, Pfizer) and Anexate (Flumazenil, 0.1mg/ml, Roche) mixture was given to the animals to antagonise the anesthetic and minimise the period of anesthesia for the animals. Ear notches were made for identification purposes and tissue was subsequently used for genotyping. The animals were then allowed a minimum recovery period of 24 hours before performing further experiments. The entire surgical procedure was performed under sterile conditions.

# In vivo µCT imaging

We scanned 2 mice. Immediately prior to each scan, animals were weighed (range of 19.9 to 24.2 gms) and anesthetized using Isoflurane (5% for induction and 1.5% for maintenance in 100% Oxygen with a flow rate of 1.5 L/min). Following anaesthesia, hair clippers were used to clear the areas near the chest and the base of the hind limb. The ECG paediatric surface electrodes (3M paediatric electrodes, USA) were placed on the skin and kept in position by medical tape. A bolus injection of 100 μl Viscover ExiTron nano 12000 (Miltenyi Biotec, Germany), an alkaline earth metal-based nanoparticular contrast agent was intravenously administered via the tail vein. This blood-pool contrast agent provides high soft tissue contrast for 4-8 hours, significantly improving visualization of the vasculature in mice with a small injection volume due to its high metal load. The nanoparticles are taken up by Kupffer cells and they circulate in the bloodstream for 8-24 hours before accumulating in the liver and spleen.^27^ The animal was placed on the mouse bed equipped with a face mask, respiratory pillow (M2M, USA) and electrical heating pad (37°C). Finally, the animal was centred in the CT field of view using laser alignment (Siemens Inveon, Germany). The field of view was selected from the aortic arch to the carotid artery bifurcations and both the internal and external carotid arteries were imaged with the parameters mentioned below.

To reduce the radiation exposure of the animals and facilitate follow-up scanning, the scanner mode was converted from conventional helical scanning to step-and-shoot mode with a settle time of 150 ms (this conversion also limited the vibration induced by the rotation of the gantry, hence minimizing motion blurring artifacts). This mode acquires images only at specific points in the cardiac cycle (triggered by R-peak in the ECG trace). The scan time for each animal was approximately 35 minutes. The animals body temperature, ECG and respiration rates were continuously monitored during the scan. The CT scan projections were reconstructed using a filtered back-projection based method modified from the Feldkamp algorithm with no down-sampling, a bilinear interpolation, and a Shepp-Logan filter using proprietary software (COBRA Exxim, Exxim Computing Corp., USA). Polynomial based soft tissue beam hardening correction was applied to the projection data for CT angiography and images were exported as DICOM files.

# Doppler velocity measurements

Each animal was anaesthetised using Isoflurane (2% inhalation) and placed on the custom bed in a supine position with its paws taped to the ECG electrodes for direct contact. The body temperature of the animal was continuously monitored using a rectal probe (Indus Instruments, Houston, TX). Hair from the neck region of the animal was removed using a chemical hair removal cream (Veet, Reckitt Benckiser North America, Inc, Persippany, NJ) and the region was covered with a layer of pre-warmed ultrasound gel (Aquasonic 100,Parker, Orange, NJ) to serve as a good coupling medium between the transducer and skin over the region of the carotid arteries. Ultrasound was performed using the high resolution imaging system Vevo 770 (Visualsonics, Toronto, Canada) RMV707B transducer with a broadband frequency of up to 45 MHz).

Initially, B-mode imaging was used for anatomically locating and identifying the entrance of the left common carotid artery (LCCA) from the aortic arch to the base of the RCCA. This was followed by Pulsed Doppler mode to measure the blood flow velocity at 1 proximal location in the RCCA immediately after the right subclavian branch that serves as the inflow velocity and another measurement made in the LCCA, towards the base of the artery. The Doppler imaging sample volume size was adjusted according to the vessel diameter and it was placed in the center of the vessels.

To obtain accurate blood velocity measurements, the vessel being imaged needs to be as parallel to the direction of the ultrasound beam as possible (i.e., the angle of incidence between the ultrasound beam and the blood flow direction in the imaged vessel should be as low as possible). Thus, the mouse bed was rotated and manipulated until beam-flow angle was minimised for Pulse Doppler measurements. The beam incidence angle for all cases was maintained below 60° to obtain accurate measurements. In most cases, it was extremely challenging to measure velocities in the internal carotid artery (ICA) and external carotid artery (ECA) branches owing to their size and their location, deep in the neck/head region and, hence, it was not performed to avoid longer scan times and inaccuracy of the measurements.

# Segmentation and 3-D reconstruction

Segmentation was performed using the open-ware image analysis software Vascular Modeling Toolkit (VMTK).^28^ The following steps describe the process. Each DICOM directory containing the CT data was loaded into VMTK to visualize the carotid anatomy in three dimensions (3-D). As the region of interest contains the pair of carotid arteries, the image datasets were cropped and the “vmtkimagevoiselector” command was used to extract the volume of interest.

The dataset was initially probed for the image intensities at various points along the length of the vessel to determine a suitable maximum and minimum threshold limit for segmentation. In regions where the grey level intensities of the lumen were similar to the surrounding soft tissue intensities (or regions where soft-tissue contrast was low), a combination of techniques was needed as thresholding alone fails to isolate the vessel from the surrounding tissue. This allowed for an accurate representation of the geometry. The “vmtklevelsetsegmentation” command was used to begin the initialisation of the model. The colliding fronts method of initialisation was used. It requires the placement of 2 seed points within the image to be segmented. A source and target seed point was placed in the carotid artery and an initial 3-D deformable model of the region between the seed points was obtained from all pixels within the threshold limits specified, by using this method of initialization. It should be noted that *in vivo* images were susceptible to contrast ‘leakiness’ in areas where nearby blood vessels might be difficult to isolate. Also, signal intensities may slightly vary along the length of the carotid artery. A global threshold might not apply to the entire length of the artery and hence local thresholds need to be specified. The model can be generated by continuous repetition of the colliding fronts method to segment smaller regions with a combination of appropriate thresholding. The segmented regions were then merged together to obtain the complete initialised model of the carotid arteries, including the carotid bifurcation.

The output from the “vmtklevelsetsegmentation” module was an image. The surface of the vessel lumen was the zero-level of that image, which was extracted by the marching cube algorithm using the “vmtkmarchingcube” command. The output of this command often results in an uneven lumen surface. This was followed by a Taubin smoothing step to even out the bumps on the surface that were physiologically unrealistic, taking care not to artificially shrink the lumen surface by over smoothing (performed by the “vmtksurfacesmoothing” module). The surface was smoothed with 100 iterations and a spatial band pass of 0.03 internal VMTK units was used in majority of the cases. When the 3-D model was determined to be sufficiently accurate, the ends were clipped (“vmtksurfaceclipper”) orthogonal to the vessel centerline and exported as an STL file to be used for CFD simulations.

# Carotid artery meshing and computational fluid dynamics

All 3-D reconstructed geometries were meshed, including the common carotid artery (inlet) and the internal (outlet 1) and external (outlet 2) carotid arteries. Flow extensions were added to each vessel branch by extruding the inlet and outlet surfaces by a factor of 1.5 and 7 times the diameter, respectively. Subsequently, the aligned meshes with the geometry extensions were imported into the final meshing interface Gambit 2.4.6 (Fluent Inc., Ansys, USA). The surface of the flow extensions was meshed with quadrilateral elements while the surface of the common carotid and carotid branches were meshed with triangular elements with a maximum edge length of 0.04 mm. A boundary layer consisting of 6 rows of prismatic elements with an initial height of 0.005 mm and growth rate of 1.2 was added to the walls of the geometry. This was done to increase the density of the mesh close to the walls in order to accurately solve the flow variables (e.g., shear stress) closer to the wall. The volume of the common carotid and internal and external branches were meshed with tetrahedral elements using the automatic meshing scheme in Gambit. Finally, the boundary zones were prescribed on the wall, inlet and outlets for the solver to correctly identify them and apply the corresponding boundary condition. A total of 22 geometries (instrumented and control carotid arteries) were meshed and mesh independence tests were carried out for each.

Each of the 22 meshed arteries was then imported into the computational fluid dynamics package Fluent version 6.3 (Ansys) to numerically solve the Navier-Stokes equations of motion for a fluid. Properties of the simulated fluid and boundary conditions were also prescribed within the model. Blood was assumed to be an incompressible Newtonian fluid with a viscosity of 3.5 mPas^29^ and density of 1050 kg/m^3^.^30^ The Newtonian fluid assumption is justified as mean shear rates are greater than 1000 1/s in the instrumented and control mouse carotid arteries, respectively, where Newtonian and non-Newtonian models of blood behave similarly ^31^. Simulations imposed a pulsatile flow of the blood at the inlet of the vessel by assigning a plug flow profile equal to the peak velocity measured at each point in the cardiac cycle, as has been reported previously^32^. The corresponding velocity wave form represented the average wave form over all cardiac cycles acquired with Doppler ultrasound. The extended inlet (described above) allowed the flow to become fully developed before entering into the actual lumen reconstruction. The outlet of the vessel had a prescribed pressure of 0 mmHg. The lumen surface was assumed to be stationary with a no-slip boundary condition. Surface monitors were created to monitor the velocity magnitude at the inlet (U) and wall shear stress throughout the simulation. Convergence was achieved when the residuals fell below 10^-5^ (m/sec) at each time step in the transient simulation. Every simulation was run over 3 cardiac cycles, where data from the first 2 cycles were excluded from the analysis to ensure independence from the initial conditions. During the 3^rd^ cardiac cycle, blood flow velocity and shear stress data were exported at every 10^th^ time step for post-processing meaning these metrics were evaluated at a total of 30 points over the cardiac cycle (the mouse heart rate was ~8 Hz, so these metrics were evaluated every 0.0042 sec over the 0.125 sec cardiac cycle).

The outputted WSS vector was then imported into a custom Matlab version R2012a (Mathworks Inc., USA) program to quantify disturbed flow, including: time-averaged wall shear stress (WSS),^33^.

References:

1	<http://www.addgene.org/>. (<http://www.addgene.org/>).

2	Openmicroscopy. (<http://www.openmicroscopy.org/>).

3	Schwarzfischer, M., Marr, C., Krumsiek, J. & Theis, F. J. (Proc. Microscopic Image Analysis with Applications in Biology, 2011).

4	Orfanidis, S. (Englewood Cliffs, NJ, 1996).

5	Altschuler, S. & Wu, L. Cellular Heterogeneity: Do Differences Make a Difference? *Cell* **141**, 559-563, doi:10.1016/j.cell.2010.04.033 (2010).

6	Huang, S. Non-genetic heterogeneity of cells in development: more than just noise. *Development* **136**, 3853-3862, doi:10.1242/dev.035139 (2009).

7	Filippi, S. *et al.* Robustness of MEK-ERK Dynamics and Origins of Cell-to-Cell Variability in MAPK Signaling. *Cell Reports* **15**, 2524-2535, doi:10.1016/j.celrep.2016.05.024 (2016).

8	Tay, S. *et al.* Single-cell NF-kappa B dynamics reveal digital activation and analogue information processing. *Nature* **466**, 267-U149, doi:10.1038/nature09145 (2010).

9	Turner, D. *et al.* Physiological levels of TNF alpha stimulation induce stochastic dynamics of NF-kappa B responses in single living cells. *Journal of Cell Science* **123**, 2834-2843, doi:10.1242/jcs.069641 (2010).

10	Cheong, R. *et al.* Transient I kappa B kinase activity mediates temporal NF-kappa B dynamics in response to a wide range of tumor necrosis factor-alpha doses. *Journal of Biological Chemistry* **281**, 2945-2950, doi:10.1074/jbc.M510085200 (2006).

11	Hoffmann, A., Levchenko, A., Scott, M. & Baltimore, D. The I kappa B-NF-kappa B signaling module: Temporal control and selective gene activation. *Science* **298**, 1241-1245, doi:10.1126/science.1071914 (2002).

12	Lipniacki, T., Paszek, P., Brasier, A., Luxon, B. & Kimmel, M. Mathematical model of NF-kappa B regulatory module. *Journal of Theoretical Biology* **228**, 195-215, doi:10.1016/j.jtbi.2004.01.001 (2004).

13	Fagerlund, R., Kinnunen, L., Kohler, M., Julkunen, I. & Melen, K. NF-kappa B is transported into the nucleus by importin alpha 3 and importin alpha 4. *Journal of Biological Chemistry* **280**, 15942-15951, doi:10.1074/jbc.M500814200 (2005).

14	Fagerlund, R., Melen, K., Kinnunen, L. & Julkunen, I. NF-kappa B is transported into the nucleus by a subset of importin a molecules. *Journal of Immunology* **178** (2007).

15	Lipniacki, T., Puszynski, K., Paszek, P., Brasier, A. & Kimmel, M. Single TNF alpha trimers mediating NF-kappa B activation: stochastic robustness of NF-kappa B signaling. *Bmc Bioinformatics* **8**, doi:10.1186/1471-2105-8-376 (2007).

16	Lagarias, J., Reeds, J., Wright, M. & Wright, P. Convergence properties of the Nelder-Mead simplex method in low dimensions. *Siam Journal on Optimization* **9**, 112-147, doi:10.1137/S1052623496303470 (1998).

17	Wang, Y. *et al.* Shear Stress Regulates the Flk-1/Cbl/PI3K/NF-kappa B Pathway Via Actin and Tyrosine Kinases. *Cellular and Molecular Bioengineering* **2**, 341-350, doi:10.1007/s12195-009-0069-3 (2009).

18	Mohan, S. *et al.* Low shear stress preferentially enhances IKK activity through selective sources of ROS for persistent activation of NF-kappa B in endothelial cells. *American Journal of Physiology-Cell Physiology* **292**, C362-C371, doi:10.1152/ajpcell.00535.2005 (2007).

19	ESI-Group. (2013).

20	Versteeg, H. & Malalasekera, W. *An Introduction to Computational Fluid Dynamics. The Finite Volume Method*. (Longman Group Ltd., 1995).

21	Vogel, M., Franke, J., Frank, W. & Schroten, H. Flow in the well: computational fluid dynamics is essential in flow chamber construction. *Cytotechnology* **55**, 41-54, doi:10.1007/s10616-007-9101-4 (2007).

22	ARMALY, B., DURST, F., PEREIRA, J. & SCHONUNG, B. EXPERIMENTAL AND THEORETICAL INVESTIGATION OF BACKWARD-FACING STEP FLOW. *Journal of Fluid Mechanics* **127**, 473-496, doi:10.1017/S0022112083002839 (1983).

23	Kaiktsis, L., Karniadakis, G. & Orszag, S. Onset of 3-dimensionality, equilibria, and early transition in flow over a backward-facing step. *Journal of Fluid Mechanics* **231**, 501-528, doi:10.1017/S0022112091003488 (1991).

24	Zarrinkoob, L. *et al.* Blood Flow Distribution in Cerebral Arteries. *Journal of Cerebral Blood Flow & Metabolism* **35**, 648-654, doi:10.1038/jcbfm.2014.241 (2015).

25	Cebral, J. R., Castro, M. A., Putman, C. M. & Alperin, N. Flow-area relationship in internal carotid and vertebral arteries. *Physiological measurement* **29**, 585-594, doi:10.1088/0967-3334/29/5/005 (2008).

26	Cheng, C. *et al.* Atherosclerotic lesion size and vulnerability are determined by patterns of fluid shear stress. *Circulation* **113**, 2744-2753, doi:10.1161/CIRCULATIONAHA.105.590018 (2006).

27	Boll, H. *et al.* Micro-CT based experimental liver imaging using a nanoparticulate contrast agent: a longitudinal study in mice. *PloS one* **6**, e25692, doi:10.1371/journal.pone.0025692 (2011).

28	Antiga, L. *et al.* An image-based modeling framework for patient-specific computational hemodynamics. *Medical & biological engineering & computing* **46**, 1097-1112, doi:10.1007/s11517-008-0420-1 (2008).

29	Trachet, B. *et al.* An integrated framework to quantitatively link mouse-specific hemodynamics to aneurysm formation in angiotensin II-infused ApoE -/- mice. *Annals of biomedical engineering* **39**, 2430-2444, doi:10.1007/s10439-011-0330-5 (2011).

30	Valentin, A., Humphrey, J. D. & Holzapfel, G. A. A finite element-based constrained mixture implementation for arterial growth, remodeling, and adaptation: theory and numerical verification. *International journal for numerical methods in biomedical engineering* **29**, 822-849, doi:10.1002/cnm.2555 (2013).

31	Weddell, J. C., Kwack, J., Imoukhuede, P. I. & Masud, A. Hemodynamic Analysis in an Idealized Artery Tree: Differences in Wall Shear Stress between Newtonian and Non-Newtonian Blood Models. *PloS one* **10**, doi:UNSP e0124575

10.1371/journal.pone.0124575 (2015).

32	Timmins, L. H. *et al.* Focal association between wall shear stress and clinical coronary artery disease progression. *Annals of biomedical engineering* **43**, 94-106, doi:10.1007/s10439-014-1155-9 (2015).

33	Rikhtegar, F. *et al.* Choosing the optimal wall shear parameter for the prediction of plaque location-A patient-specific computational study in human left coronary arteries. *Atherosclerosis* **221**, 432-437, doi:10.1016/j.atherosclerosis.2012.01.018 (2012).
